# Influence of dextrose monohydrate on the optical properties and adsorption activity of Ni_0.6_Zn_0.2_Sb_0.2_Fe_2_O_4_ nanoferrites: towards multifunctional applications

**DOI:** 10.1039/d5ra08391e

**Published:** 2026-01-21

**Authors:** Nourhan Mohamed Gaber, Leen W. El Khatib, Amani Aridi, Alaa M. Abdallah, Gehan M. El-Subruiti, Mirna Omar, Sarah Omar, Ramadan Awad

**Affiliations:** a Department of Medical Laboratories, Faculty of Applied Health Science Technology, Pharos University in Alexandria Canal El Mahmoudia Street, Beside Green Plaza Complex Alexandria 21648 Egypt nourhan.gaber@pua.edu.eg; b Department of Chemistry, Faculty of Science, Beirut Arab University Beirut Lebanon; c Public Health Department, Faculty of Health Sciences, Modern University for Business and Science Beirut Lebanon; d Physics Department, Faculty of Science, Beirut Arab University Beirut Lebanon; e Chemistry Department, Faculty of Science, Alexandria University Alexandria Egypt; f Department of Mechanical Engineering, Universiti Teknologi PETRONAS (UTP) 32610 Bandar Seri Iskandar Perak Darul Ridzuan Malaysia; g Physics Department, Faculty of Science, Alexandria University Alexandria Egypt; h Department of Basic Sciences, Faculty of Computer Science and Artificial Intelligence, Pharos University in Alexandria Alexandria Egypt

## Abstract

Dyes are noxious substances that are frequently found in industrial effluent. Metal ferrites are magnetic nano-adsorbents and may be affordable and effective in removing pollutants. Capping these nanoparticles increases their stability and prevents them from aggregating. In this investigation, Ni_0.6_Zn_0.2_Sb_0.2_Fe_2_O_4_ (Ni_0.6_Zn_0.2_Sb_0.2_F) nanoparticles were capped with different concentrations of dextrose monohydrate (*D*), forming Ni_0.6_Zn_0.2_Sb_0.2_FD *via* the co-precipitation method. The optical properties of the samples were investigated by utilizing UV-vis spectroscopy and photoluminescence spectroscopy (PL) to unveil their multifunctional potential. Several parameters were calculated, such as the band gap energy, which varied between 2.71 eV and 3.05 eV. Furthermore, the interstitial defects and the recombination process of electrons were also determined using PL. The adsorption activity of the synthesized Ni_0.6_Zn_0.2_Sb_0.2_FD was investigated against the removal of methyl violet (MV). The best removal efficiency was achieved by Ni_0.6_Zn_0.2_Sb_0.2_FD_10_ with a value of 78.5%, and maximum adsorption capacity (*q*_max_), *q*_max_ = 75.5 mg g^−1^. This was attained by studying several parameters, including adsorbent dosage, MV concentration, pH, temperature, and time. The optimum experimental conditions were obtained at 10 ppm MV, pH = 7, and temperature 25 °C. The adsorption process was found to follow a pseudo-second-order model, and the experimental data fit well with the Temkin non-linear isotherm models.

## Abstract 2

Dextrose monohydrate-capped Ni_0.6_Zn_0.2_Sb_0.2_Fe_2_O_4_ offers tunable optical band gaps and high methyl violet adsorption, showing significant potential for synergistic multifunctional optoelectronic and environmental applications.

## Graphical abstract

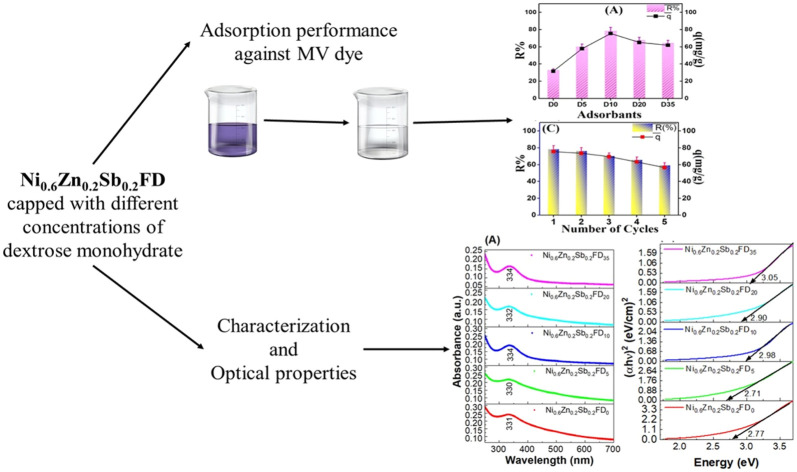

## 1. Introduction

Water supplies are under threat owing to over-exploitation by humans and pollution from industrial waste and agricultural runoff. Dyes, petrochemicals, heavy metals, and pharmaceutical effluents permeate the hydrosphere annually, causing various environmental impacts. According to research, dyes enter the environment at a rate of 70 000 to 2 00 000 tons per year.^1^ They are classified based on their chemical structure and application technique. Methyl violet (MV) is a triphenylmethane dye, having three aryl groups attached to a nitrogen atom and one or two methyl groups with a long half-life, resulting in its vivid coloration. Furthermore, MV is a basic dye due to the positive charge on its amino groups and is composed of tetramethyl, pentamethyl, and hexamethyl pararosaniline chlorides.^2^ This latter molecule, commonly known as crystal violet or MV10B, is the principal representative of the three dyes. In contrast, the penta-methylated molecule dominates in MV2B. The color of MV varies based on the ratio of each component.^3^ MV is often utilized in several sectors, including paper, textile, printing, and leather, despite its carcinogenicity, non-biodegradability, toxicity, and mutagenicity.^4^ Its bio-resistant existence in water causes turbidity and reduced light penetration, inhibiting the photosynthetic processes of aquatic plants.^5^ Moreover, this MV discharge can impair human health by negatively impacting the respiratory system, digestive system, and kidneys. Also, it causes other symptoms, including hypertension, jaundice, accelerated heartbeat, vomiting, cyanosis, ocular discomfort, and shock.^4^

Removing synthetic dyes from industrial effluent is problematic due to their complicated aromatic structure and lack of biodegradability. Implementing proper techniques to eliminate MV from dye effluent is crucial for maintaining water quality and protecting human health. Several sanitation treatment technologies are often used to remove dyes from wastewater, such as physical, physicochemical, and chemical methods. These methods include membrane separation, sedimentation, coagulation, flocculation, adsorption, solvent extraction, photocatalytic oxidation, Fenton oxidation, and electrochemical approaches. Moreover, biological approaches include aerobic and anaerobic microbial decomposition, as well as the application of enzymes. Chemical and biological procedures are effective in removing hues, but they require specific tools and are energy-intensive. Additionally, they typically create significant amounts of byproducts.^6^ The physical pollutant removal approach is green and effective as it removes the pollutants without generating by-products. Adsorption offers merits over other techniques owing to its easy implementation, simple design, significant regeneration potential, diversified material sources, strong performance, and affordability. This method is ideal for treating polluted waters as it does not need prior treatment.^7^

Spinel ferrite (SFs) nanoparticles are metallic oxides, defined by the generic chemical formula AB_2_O_4_, where the metal cations denoted by A and B are located at tetrahedral and octahedral sites, respectively. The anionic oxygen in AB_2_O_4_ creates dense face-centered cubic (FCC) packing. Spinel ferrites have gained popularity due to their high adsorption capacity, unique magnetic and electrical characteristics, and large surface area-to-volume ratio, making them a popular choice for wastewater treatment.^8^ According to the cations' distribution in the spinel lattice, SFs can be further categorized into three groups based on the cation distribution: normal, inverse, and mixed spinel ferrites. In normal spinel ferrites, the divalent ions are present in the tetrahedral site, while the trivalent ions are present in the octahedral site as ZnFe_2_O_4_. Whereas, in inverse spinel ferrites, the divalent ions are present in the octahedral site, while the trivalent ions are distributed equally between the tetrahedral and the octahedral sites as NiFe_2_O_4_. In mixed spinel ferrites, the divalent and trivalent ions are both present in the tetrahedral and octahedral sites, such as NiZnFe_2_O_4_. The mixed spinel structure has significant properties combined from both normal and inverse ones, such as high electrical resistivity, high magnetic permeability, high saturation magnetization, high corrosion resistance, low dielectric constant, and low magnetic coercivity.^9^

Despite the superior magnetic behaviour of SFs, agglomeration occurs between their particles, causing a lowering in active adsorption sites; thus, capping agents are used such as polysaccharides, polymers, amino acids, proteins, dendrimers, cyclodextrins, organic ligands, surfactants, and bio-extracts.^10,11^ One of the most used capping agents is the dextrose monohydrate (*D*). *D* is a carbohydrate derivative, specifically a monosaccharide characterized by its availability, solubility in water, and low price.^12^ The amphiphilic *D* molecules, which have polar and nonpolar heads, react with metals of SFs and with the surrounding environment, respectively. By forming covalent bonds with the SFs, *D* causes steric hindrance, resulting in increased stability in the adsorbent.^13^

Previously, Khatib *et al.*^12^ fabricated an eco-friendly uncapped and capped Ni_0.6_Zn_0.2_Sb_0.2_FD relying on the characteristics of dextrose monohydrate at different concentrations. The physical and magnetic properties of the synthesized samples were published in a previous work, including the XRD, TEM, EDX, and VSM characterizations. Since the fabricated nanoferrites have the potential to be applied in dye removal, this study was performed to investigate the optical properties of the uncapped and capped Ni_0.6_Zn_0.2_Sb_0.2_FD using UV-vis and PL spectroscopies. Besides, the adsorption activity of these samples was tested. A full elimination study was conducted to establish the best conditions for MV removal, such as the effect of pH, initial MV concentrations, adsorbent dosage, and the effect of temperature. The proposed adsorption pathway was predicted through a comparison between FTIR spectra before and after MV removal. Moreover, kinetics, isotherms, and thermodynamic adsorption studies were employed. Eventually, a reusability test was held to assess the stability of the capped Ni_0.6_Zn_0.2_Sb_0.2_FD.

## 2. Materials and methods

### 2.1. Chemicals and materials

Iron(iii) chloride hexahydrate (FeCl_3_·6H_2_O ≥ 99%, honeywell Fluka), nickel(ii) chloride hexahydrate (NiCl_2_·6H_2_O ≥ 98%, Sigma Aldrich), zinc chloride (ZnCl_2_ ≥ 98%, Sigma Aldrich), antimony(iii) chloride (SbCl_3_ ≥ 99%, Sigma Aldrich), sodium hydroxide pellet (NaOH ≥ 99%, Chem-Lab) and dextrose monohydrate (C_6_H_12_O_6_·H_2_O, Alpha Chemika). The solvents used throughout the synthesis process are deionized water and ethanol. All chemicals used were analytical grade and did not require additional purification. Furthermore, deionized water was used throughout the experiment.

### 2.2. Preparation of uncapped and capped Ni_0.6_Zn_0.2_Sb_0.2_FD

The co-precipitation method was adopted to synthesize uncapped and capped Ni_0.6_Zn_0.2_Sb_0.2_FD with different concentrations of *D*. A 1 M solution of each nickel-, zinc-, and antimony chloride was prepared separately by dissolving in deionized water under stirring to ensure full dissolving, then mixed. This was followed by the progressive addition of different concentrations of dextrose monohydrate (0 M, 0.05 M, 0.1 M, 0.2 M, and 0.35 M) dropwise to the mixtures. The final mixtures were stirred for 30 minutes, followed by titration with 3 M NaOH to adjust the pH value to 12. After that, the mixtures were heated under continuous stirring for 2 hours at 80 °C. The mixtures were then washed with a mixture of 75% deionized water and 25% ethanol to adjust the pH value to 7. Furthermore, the obtained samples were dried in an oven for 16 hours at 100 °C, ground into powder, and then calcined for 2 hours at 750 °C. The samples will be named as follows: Ni_0.6_Zn_0.2_Sb_0.2_FD_0_, Ni_0.6_Zn_0.2_Sb_0.2_FD_5_, Ni_0.6_Zn_0.2_Sb_0.2_FD_10_, Ni_0.6_Zn_0.2_Sb_0.2_FD_20,_ and Ni_0.6_Zn_0.2_Sb_0.2_FD_35_ for *D* concentrations of 0, 0.05, 0.1, 0.2, and 0.35 M, respectively.

### 2.3. Optical studies

Optical studies were carried out by a UV-vis Jasco V-670 spectrophotometer in the range 250–700 nm. The Jasco FP-8600 spectrofluorometer was utilized to investigate the photoluminescence spectroscopy (PL) studies. In these characterization techniques, 4 mg of the uncapped and capped Ni_0.6_Zn_0.2_Sb_0.2_FD were dissolved in 40 mL of HCl (2 M) and sonicated for 15 minutes. Then, the measurement was done by transforming 5 mL of the solution into a quartz cuvette.

### 2.4. Adsorption studies

The MV dye adsorption experiments were performed in batch mode. Different amounts of Ni_0.6_Zn_0.2_Sb_0.2_FD_10_ (0.01–0.04 g) were added to 20 mL solutions of MV dye with initial concentrations ranging from 10 to 50 mg L^−1^. The pH of the solution was adjusted between 3 and 11 using 0.1 M HCl and 0.1 M NaOH solutions, and the temperature during adsorption varied from 25 to 55 °C. Samples were taken at specific time intervals, filtered, and the remaining MV concentration was determined at 590 nm using a vis-spectrophotometer. Moreover, the point of zero charge (pH_pzc_) was determined using the Potentiometric Mass Titration (PMT) technique. The removal percentage of the dye and the adsorption capacity *q*_*t*_ were calculated using the following eqn (1) and (2):1
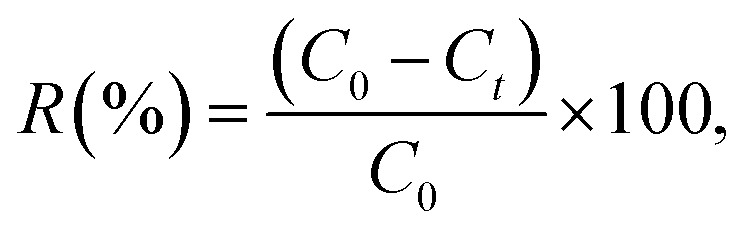
2
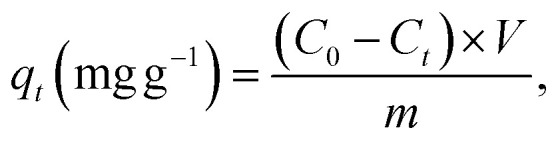
where *C*_0_ and *C*_*t*_ denote the concentrations of MV dye at *t*_0_ and at time *t*_*t*_, respectively. The volume of the MV solution is indicated by *V* in L, and *m* represents the mass of Ni_0.6_Zn_0.2_Sb_0.2_FD in g.

### 2.5. Recyclability test

The reusability of the Ni_0.6_Zn_0.2_Sb_0.2_FD_10_ was determined during five consecutive MV removal processes. After each elimination process, the Ni_0.6_Zn_0.2_Sb_0.2_FD_10_ adsorbent was separated *via* its exhibited magnetic properties, rinsed with distilled water, and oven-dried at 60 °C for an overnight period. The resurrected adsorbent was subsequently used in subsequent removal operations.

## 3. Results and discussion

### 3.1. Characterization of the adsorbent

The samples were characterized, in a previously published work, using X-ray powder diffraction (XRD), transmission electron microscopy (TEM), energy dispersive X-ray analysis (EDX), and vibrating sample magnetometer (VSM). The results are summarized in Table 1.^12^

**Table 1:** Summary of the results obtained from different characterization techniques^12^

Method of characterization	Parameter	Ni_0.6_Zn_0.2_Sb_0.2_FD_0_		Ni_0.6_Zn_0.2_Sb_0.2_FD_5_	Ni_0.6_Zn_0.2_Sb_0.2_FD_10_		Ni_0.6_Zn_0.2_Sb_0.2_FD_20_	Ni_0.6_Zn_0.2_Sb_0.2_FD_35_	
XRD	*a* (Å)	8.361		8.360	8.360		8.357	8.356	
	Phase percentage of ferrite (%)	91.482		95.149	94.640		97.460	99.479	
	Phase percentage of hematite (%)	8.518		4.851	5.360		2.540	0.521	
	*D* (nm)	18.334		20.543	22.785		17.692	16.367	
TEM	*D*_TEM_ (nm)	15.402		16.516	16.773		15.320	15.258	
VSM	*M*_s_ (emu g^−1^)	35.836		39.757	39.416		40.813	39.503	
	*H*_c_ (G)	25.981		36.845	39.501		35.644	29.814	
EDX		Average	Theoretical		Average	Theoretical		Average	Theoretical
	Ni:Fe	0.299	0.3		0.279	0.3		0.300	0.3
	Zn:Fe	0.084	0.1		0.087	0.1		0.092	0.1
	Sb:Fe	0.104	0.1		0.104	0.1		0.116	0.1
	O:Fe	2.292	2		2.089	2		2.811	2

The lattice parameter ‘*a’* is found to decrease with the increase in the dextrose monohydrate capping agent. This decrease in the lattice parameter with the presence of dextrose monohydrate is due to the improved crystallinity and reduction of nuclei of nanoferrites.^12^ However, the crystallite size increases with the increase in dextrose monohydrate capping agent up to a maximum of 22.785 nm for sample Ni_0.6_Zn_0.2_Sb_0.2_FD_10_. Further increase in dextrose monohydrate concentration leads to a decrease in the crystallite size. This is explained on the basis that a concentration of 0.05 M and 0.1 M of dextrose monohydrate improves the crystallite growth in the synthesized samples, while the decrease obtained at higher dextrose monohydrate concentrations is a result of the excess amount of dextrose monohydrate absorbed on the surface of the nanoferrites.^14,15^ Regarding the phase percentage, it is noted that the phase percentage of the nanoferrites increases while the phase percentage of hematite (α-Fe_2_O_3_) decreases with the increase in dextrose monohydrate concentration. This proves that the capping agent improves the formation of the Ni_0.6_Zn_0.2_Sb_0.2_FD phase and retards the formation of the α-Fe_2_O_3_ phase.^16,17^ The particle size obtained from TEM increases with the increase in the capping agent concentration up to a maximum of 16.773 nm for a dextrose monohydrate concentration of 0.1 M. Further increases in capping agent concentration cause a decrease in the particle size. The EDX patterns proved the presence of Ni, Zn, Sb, Fe, and O in all samples, in addition to carbon (C) in dextrose monohydrate capped samples. Moreover, the atomic percentage of the different elements and their average ratios obtained from the different regions were similar and comparable to the theoretical ones. The obtained ratios are displayed in Table 1. The bulk dextrose monohydrate loading calculated from EDX is found to be in the range 5–8%, while the surface loading obtained from XPS is found to be in the range 15–17% according to the equations (a–c)^18,19^ present below. The higher loading value obtained from XPS is explained on the basis that XPS is a surface-sensitive quantitative spectroscopic technique that measures the very topmost atoms. Since dextrose monohydrate is used as a capping agent, it forms a thin protective layer around the surface of the nanoferrites rather than being a bulk material. The saturation magnetization *M*_s_ values determined from VSM increase with the increase in dextrose monohydrate concentration to a maximum of 40.813 emu g^−1^ for Ni_0.6_Zn_0.2_Sb_0.2_FD_20_ sample. The decrease in hematite percentage and the variation in the crystallite size are the causes of the increase in *M*_s_ values. This proves the change in magnetic behaviour.^20^ The increase in dextrose monohydrate concentration causes an increase in the coercivity *H*_c_ values to a maximum of 39.501 G for the Ni_0.6_Zn_0.2_Sb_0.2_FD_10_ sample. Further increase in capping agent concentration leads to a decrease in *H*_c_ values.3
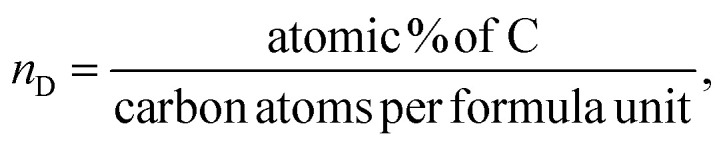
4
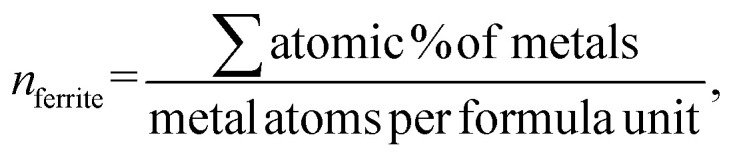
5
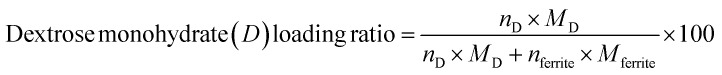


### 3.2. UV-vis spectroscopy analysis

**Fig. 1:**
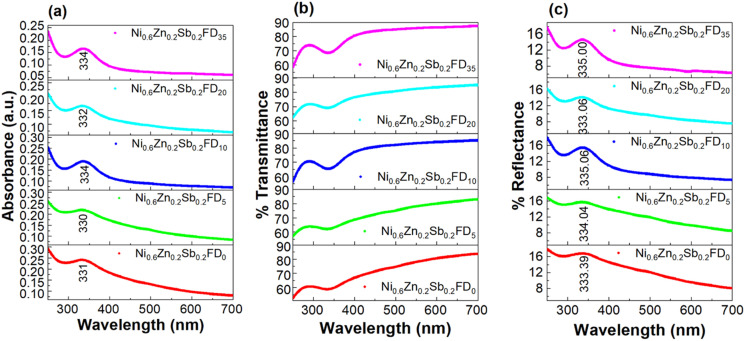
The absorbance (a), transmittance (b), and reflectance (c) spectra of the uncapped and capped Ni_0.6_Zn_0.2_Sb_0.2_FD.

To measure the optical properties of the synthesized nanoparticles, UV-vis spectroscopy was used. The absorbance, transmittance, and reflectance spectra of uncapped and capped Ni_0.6_Zn_0.2_Sb_0.2_FD are represented in Fig. 1(a–c). A high absorption peak is observed in all samples in the range (330–334 nm). The variation in this peak is related to the variation in energy bandgap (*E*_g_).^21^ Previous studies investigated the UV-vis spectroscopy of NiFe_2_O_4_ nanoparticles, where an absorption peak is observed at 338 nm by Kumari *et al.*^22^ On the other hand, the absorption peak of ZnFe_2_O_4_ is at 433 nm as investigated by Talebi *et al.*^23^ The absorbance peak of Ni_0.5_Zn_0.5_Fe_2_O_4_ is at 376 nm, as shown by Chehade *et al.*^24^ However, the transmittance follows an opposite trend to that of absorbance, where the transmittance is minimum in the UV region and increases with the increase in wavelength, so that the maximum is obtained in the visible region. The reflectance, represented in Fig. 1(c), follows the same trend as that of absorbance. The maximum reflectance appears in the UV region, while the minimum occurs in the visible region. Upon the increase in dextrose monohydrate capping agent concentration, the reflectance decreases. Therefore, the synthesized nanoparticles can be utilized in transparent electrodes and optoelectronic devices.^21^

**Fig. 2:**
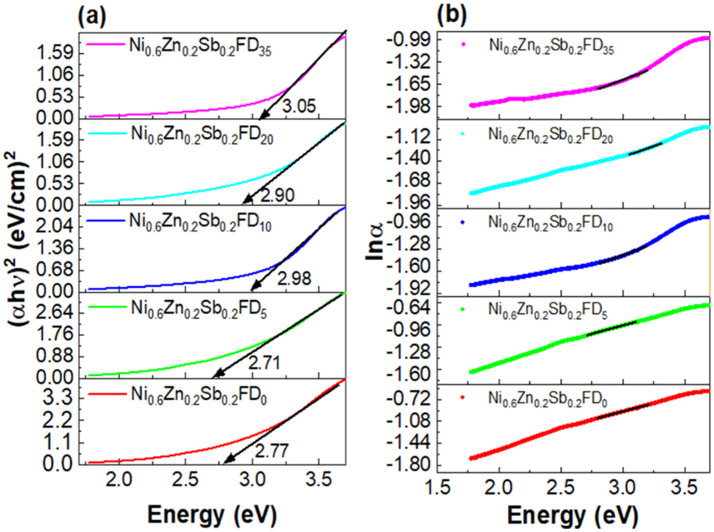
Tauc plots (a) and Urbach energy (b) of the uncapped and capped Ni_0.6_Zn_0.2_Sb_0.2_FD.

The direct energy gap of the synthesized uncapped and capped nanoferrites is extracted from Tauc plots, provided in Fig. 2(a), and calculated according to the following eqn (6):^21^

6 (*αhν*)^*n*^ = *B*(*hν* − *E*_g_),

where *α* is the absorption coefficient 
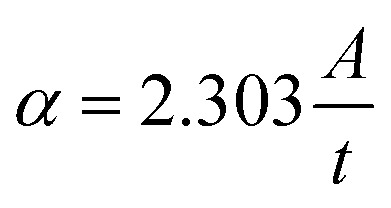
*A* is the absorbance, and *t* is the sample thickness.^25^*hν* is the excitation energy, *B* is a constant, *E*_g_ is the energy gap, and *n* is a constant that depends on the type of transition. Here, *n* is taken to be equal to 2 for the allowed direct transition. The crystallite size, the lattice parameters, the degree of stoichiometry, the substituted elements, the cation distribution, and the presence of impurities are factors that affect the value of *E*_g_.^21^ The *E*_g_ values decreased with the addition of dextrose monohydrate in sample Ni_0.6_Zn_0.2_Sb_0.2_FD_5_ till 2.71 eV and then increased with the increase in dextrose monohydrate concentration till reaching a value of 3.05 eV for Ni_0.6_Zn_0.2_Sb_0.2_FD_35_ sample. Thus, *E*_g_ values are not greatly affected by the concentration of the capping agent. The variation in the *E*_g_ values is due to the perturbations occurring in the electronic structure upon introducing the dextrose monohydrate as a capping agent.

**Fig. 3:**
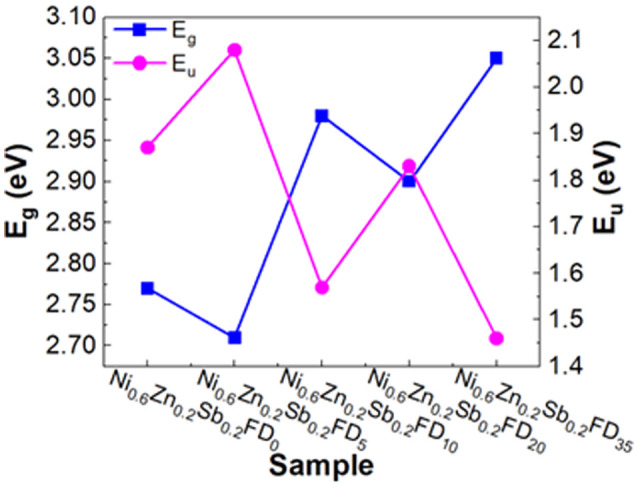
The variation of *E*_g_ and *E*_u_ of Ni_0.6_Zn_0.2_Sb_0.2_FD capped with different concentrations of dextrose monohydrate.

To determine the band tail width of the localized states, the Urbach energy was calculated using eqn (7):7
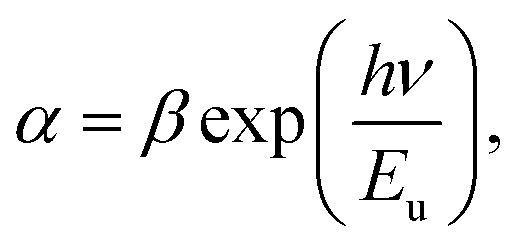
where *β* is a constant and *E*_u_ is the Urbach energy, which measures the disorder in the structure of the synthesized nanoferrites. The *E*_u_ values are obtained by plotting ln(*α*) *versus hν* as shown in Fig. 2(b), where *E*_u_ is equal to the reciprocal of the slope. As shown in Fig. 3, the *E*_u_ values vary between 1.46 and 2.08 eV in the presence of dextrose monohydrate capping agent. The addition of dextrose monohydrate capping agent causes a decrease in *E*_u_ values except for Ni_0.6_Zn_0.2_Sb_0.2_FD_5_. Therefore, the addition of the capping agent leads to a decrease in defects and disorder represented by the decrease in *E*_u_ values and, consequently, an increase in *E*_g_ values, except in the Ni_0.6_Zn_0.2_Sb_0.2_FD_5_ sample, as displayed in Fig. 3. Thus, *E*_u_ and *E*_g_ have an inverse behaviour, following the general trend. Hence, the synthesized samples, especially Ni_0.6_Zn_0.2_Sb_0.2_FD_5_, with the lowest *E*_g_, are suitable for photocatalytic activity and optoelectronic devices.^26–28^

The skin depth *δ*, the optical density *D*_opt_, and the extinction coefficient *k* of the synthesized nanoferrites are calculated according to the following eqn (8)–(10).^25,29,30^8
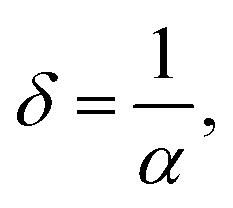

9 *D*_opt_ = *α* × *t*, and

**Fig. 4:**
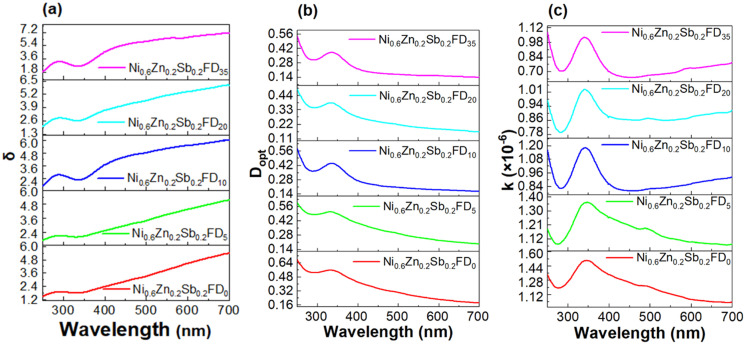
The variation of (a) *δ*, (b) *D*_opt_, and (c) the extinction coefficient as a function of the wavelength of the uncapped and capped Ni_0.6_Zn_0.2_Sb_0.2_FD.

10
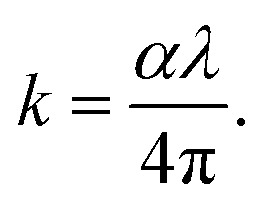
where *λ* is the wavelength. As depicted in Fig. 4(a and b), the skin depth of the synthesized nanoferrites increases, while the optical density decreases upon the increased addition of dextrose monohydrate capping agent. This variation in optical density is due to a decrease in absorption.^30^ The extinction coefficient is an indication of the amount of energy dissipated by the synthesized nanoferrites due to the absorption and scattering phenomena.^31^ As shown in Fig. 4(c), the extinction coefficient decreases with the increase in the wavelength, and the highest value of the extinction coefficient is obtained in the wavelength range 340–345 nm. This decrease in *k* upon the increase in wavelength indicates the decrease in the fraction of light lost as a result of absorbance and scattering in this region.^32^ Also, the reduction of *k* in the visible region proves the elevation of transmittance in this region.^24^ It is also observed that upon the addition of a dextrose monohydrate capping agent and the increase in its concentration, the extinction coefficient decreases. These results show that the samples are suitable for application in optoelectronic devices.^33^

**Fig. 5:**
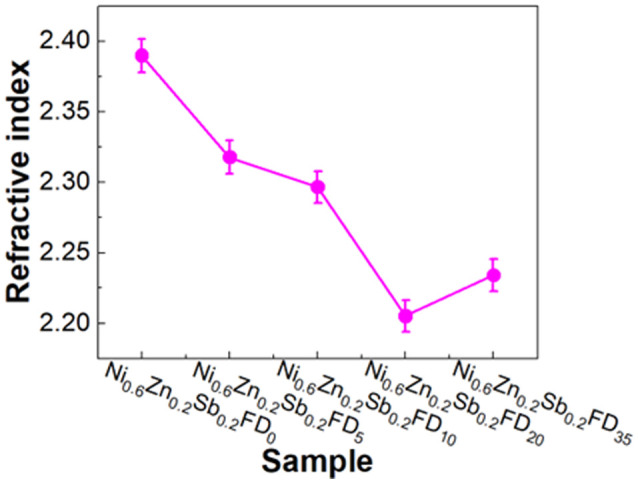
The refractive index for the uncapped and capped Ni_0.6_Zn_0.2_Sb_0.2_FD.

The refractive index *n* is one of the most fundamental parameters for materials applicable in optical domains such as optical sensors, switches, modulators, and optical filters. To calculate the refractive index, eqn (11) is used:^33^11
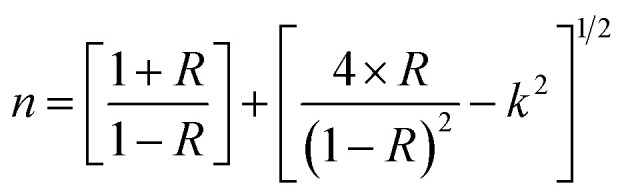
where *R* is the reflectance and *k* is the extinction coefficient. The refractive index values obtained are presented in Fig. 5. The refractive index calculated using the mentioned method varies between 2.21 and 2.39. The refractive index value decreases upon the addition of different concentrations of the capping agent and is comparable to those obtained in the literature.^33,34^ This indicates that the synthesized Ni_0.6_Zn_0.2_Sb_0.2_FeD nanoferrites can be used in optoelectronic, solar, and technological applications.^33,34^

The real dielectric constant *ε*_r_, the imaginary dielectric constant *ε*_*i*_, and the dielectric loss factor tan *δ*, which indicates the depletion of the electrical energy, can be determined from the refractive index and the extinction coefficient according to the following eqn (12)-(14):^30,33^

12 *ε*_r_ = *n*^2^ − *k*^2^,

13 *ε*_i_ = 2*nk*,

14
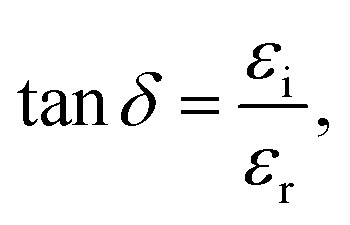


**Fig. 6:**
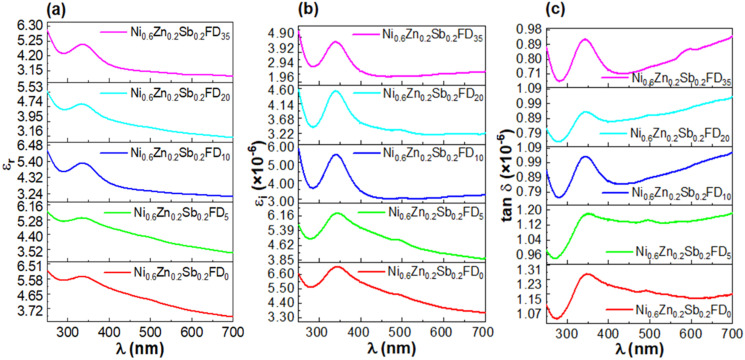
The variation of the real dielectric constant (a), the imaginary dielectric constant (b), and the dielectric loss factor (c) *versus* wavelength of the uncapped and capped Ni_0.6_Zn_0.2_Sb_0.2_FD.

*ε*_r_ shows the extent to which the material will reduce the speed of light, while *ε*_i_ indicates how the dielectric material absorbs energy from an electric field as a result of the dipole motion.^33^ The variation of *ε*_r_, *ε*_i,_ and tan *δ* as a function of wavelength is displayed in Fig. 6(a–c), respectively. As observed, *ε*_r_, *ε*_i_, and tan *δ* values are almost decreasing with the increase in the wavelength. At low wavelength, a maximum is observed in the range 334–336 nm for *ε*_r_, while the maximum of *ε*_i_ is found to be in the range 337–342 nm, and that of tan *δ* is in the range 343–348 nm. The high value of *ε*_r_ and *ε*_i_ at low wavelengths signifies an insulating behaviour.^33^ The addition of dextrose monohydrate capping agent and the increase in its concentration cause a decrease in *ε*_r_, *ε*_i_, and tan *δ*.

The optical conductivity *σ*_opt_ and electric conductivity *σ*_e_ are calculated using the following eqn (15) and (16):^25^15
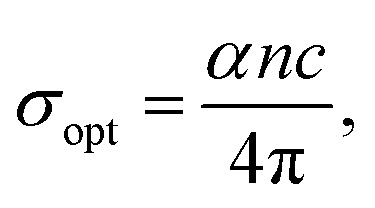
16
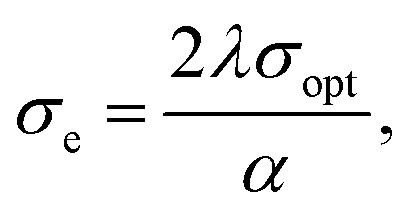
where *c* is the speed of light in a vacuum.

**Fig. 7:**
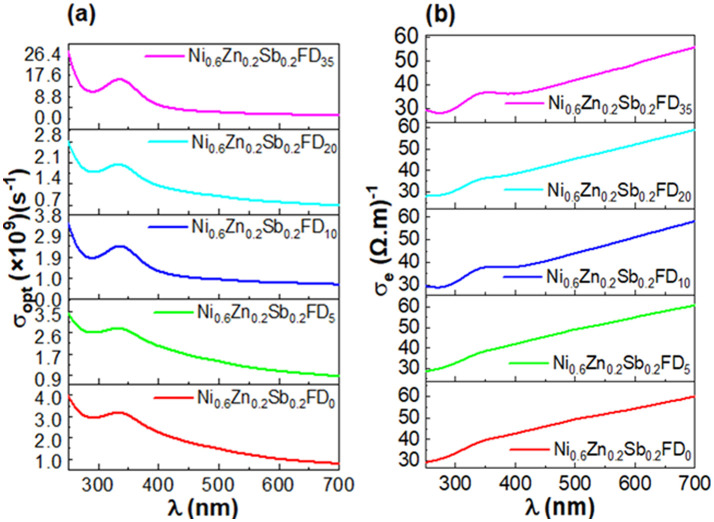
The variation of *σ*_opt_ (a) and *σ*_e_ (b) as a function of the wavelength of the uncapped and capped Ni_0.6_Zn_0.2_Sb_0.2_FD.

The variation of *σ*_opt_ and *σ*_e_ as a function of the wavelength is present in Fig. 7(a and b). It is clear that *σ*_opt_ attained a maximum in the wavelength range 331–335 nm, then decreased with the increase in wavelength to attain a minimum in the visible region. However, *σ*_e_ increases with the increase in wavelength. *σ*_opt_ is directly proportional to the refractive index and the absorption coefficient. The increase in *σ*_opt_ in the wavelength range 331–335 nm is due to the increase in the absorption coefficient in this range and the increase in the excitation of electrons.^29^ Also, *σ*_e_ values are much lower than *σ*_opt_ as a result of a deficiency in the energy needed for the free carriers to jump the potential barrier level.^33^ This renders the synthesized nanoferrites as potential candidates in optoelectronic devices.^33^

### 3.3. Determination of *E*_0_, *E*_d_, *S*_0_, *λ*_0_, *ε*_1_ and *γ*

Wemple and Di Domenico's relationship eqn (17) expresses the dispersion of the refractive index in the synthesized nanoferrites using the concept of the single oscillator:^33^17
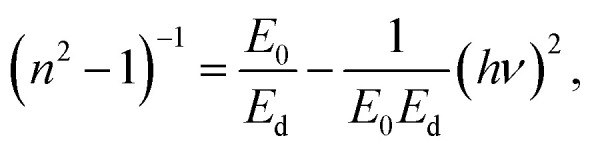
where *E*_0_ is the oscillator energy and *E*_d_ is the dispersion energy, which measures the average strength of interband optical energy.

**Fig. 8:**
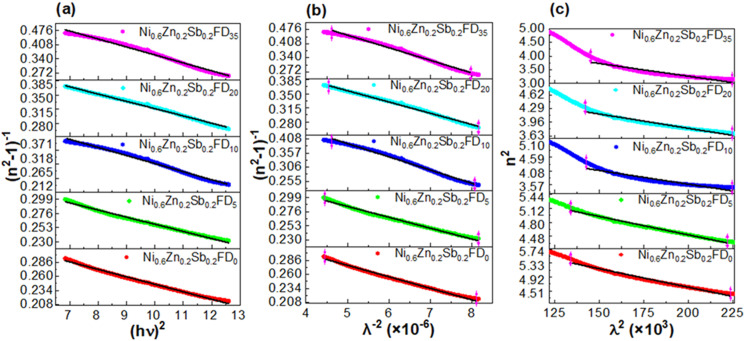
The linear fit of (a) (*n*^2^ − 1)^−1^*versus* (*hν*)^2^, (b) (*n*^2^ − 1)^−1^*versus λ*^−2^, and (c) *n*^2^*versus λ*^2^ of the uncapped and capped Ni_0.6_Zn_0.2_Sb_0.2_FD.

Fig. 8(a) displays the linear fit of (*n*^2^ − 1)^−1^*versus* (*hν*)^2^. The obtained values are listed in Table 2, where *E*_0_ and *E*_d_ can be determined using the slope 
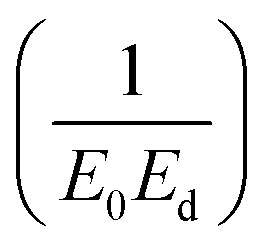
 and the intercept 
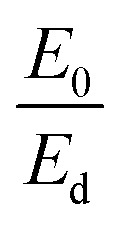
. *E*_0_ is related to the optical band gap energy by the following equation: *E*_0_ ≈ *γE*_g_. Where *γ* is a coefficient of proportionality and is approximately equal to 2. The slight variation between *E*_g_ obtained from Tauc plot and *E*_g_ calculated from Wemple and Di Domenico's relation is due to the difference in the region used for performing the calculation.^35^ The transparent region of the spectrum is used for calculation in Wemple and Di Dominco's relation, while in Tauc plot, the absorption region is used.

**Table 2:** The values of the optical parameters of the uncapped and capped Ni_0.6_Zn_0.2_Sb_0.2_FD

Sample	*E*_0_ (eV)	*E*_d_ (eV)	*S*_0_ (m^−2^)	*λ*_0_ (nm)	*ε*_1_	*γ*
Ni_0.6_Zn_0.2_Sb_0.2_FD_0_	5.28	13.80	4.71 × 10^−5^	236	6.80	1.09 × 10^−5^
Ni_0.6_Zn_0.2_Sb_0.2_FD_5_	5.69	15.22	5.63 × 10^−5^	218	6.20	8.44 × 10^−6^
Ni_0.6_Zn_0.2_Sb_0.2_FD_10_	4.53	7.28	2.09 × 10^−5^	275	5.62	1.01 × 10^−5^
Ni_0.6_Zn_0.2_Sb_0.2_FD_20_	5.25	10.57	3.57 × 10^−5^	237	5.18	6.85 × 10^−6^
Ni_0.6_Zn_0.2_Sb_0.2_FD_35_	4.41	5.95	1.65 × 10^−5^	284	5.12	9.30 × 10^−6^

The single term Sellmeier oscillation expresses the variation of the refractive index as a function of wavelength according to the following eqn (18):^35^18
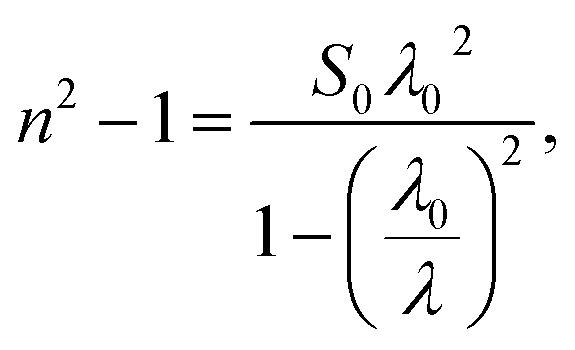
where *S*_0_ is the average oscillator strength, *λ*_0_ is the average oscillator wavelength, and *λ* is the wavelength of the incident light. The plot of (*n*^2^ − 1)^−1^*versus λ*^−2^ is present in Fig. 8(b). The values of *S*_0_ and *λ*_0_ are obtained from the slope and intercept of the curve present in Fig. 8(b) and are listed in Table 2. As shown, *S*_0_ and *λ*_0_ values are found to vary with the addition of a dextrose monohydrate capping agent and the increase in its concentration. The highest value of *λ*_0_ in the presence of the capping agent is found to be 284 nm for Ni_0.6_Zn_0.2_Sb_0.2_FD_35_.

The lattice dielectric constant *ε*_1_ is determined to obtain more information on the optical properties of the synthesized nanoparticle using the following eqn (19):^33^

19 *n*^2^ = *ε*_1_ − *γλ*^2^

where *γ* is a constant. *ε*_1_ value is the intercept with the vertical axis obtained by extrapolating the linear part toward a shorter wavelength, as present in Fig. 8(c). The values are tabulated in Table 2. As shown in Table 2, *ε*_1_ values decrease with the addition of a dextrose monohydrate capping agent and with the increase in its concentration.

The calculated parameters present in Table 2 are found to be influenced by the concentration of the capping agent, indicating that the capping agent helps improve the optical properties of the synthesized samples and consequently boosts their integration in optoelectronic applications.^33^

### 3.4. Photoluminescence spectroscopy (PL)

**Fig. 9:**
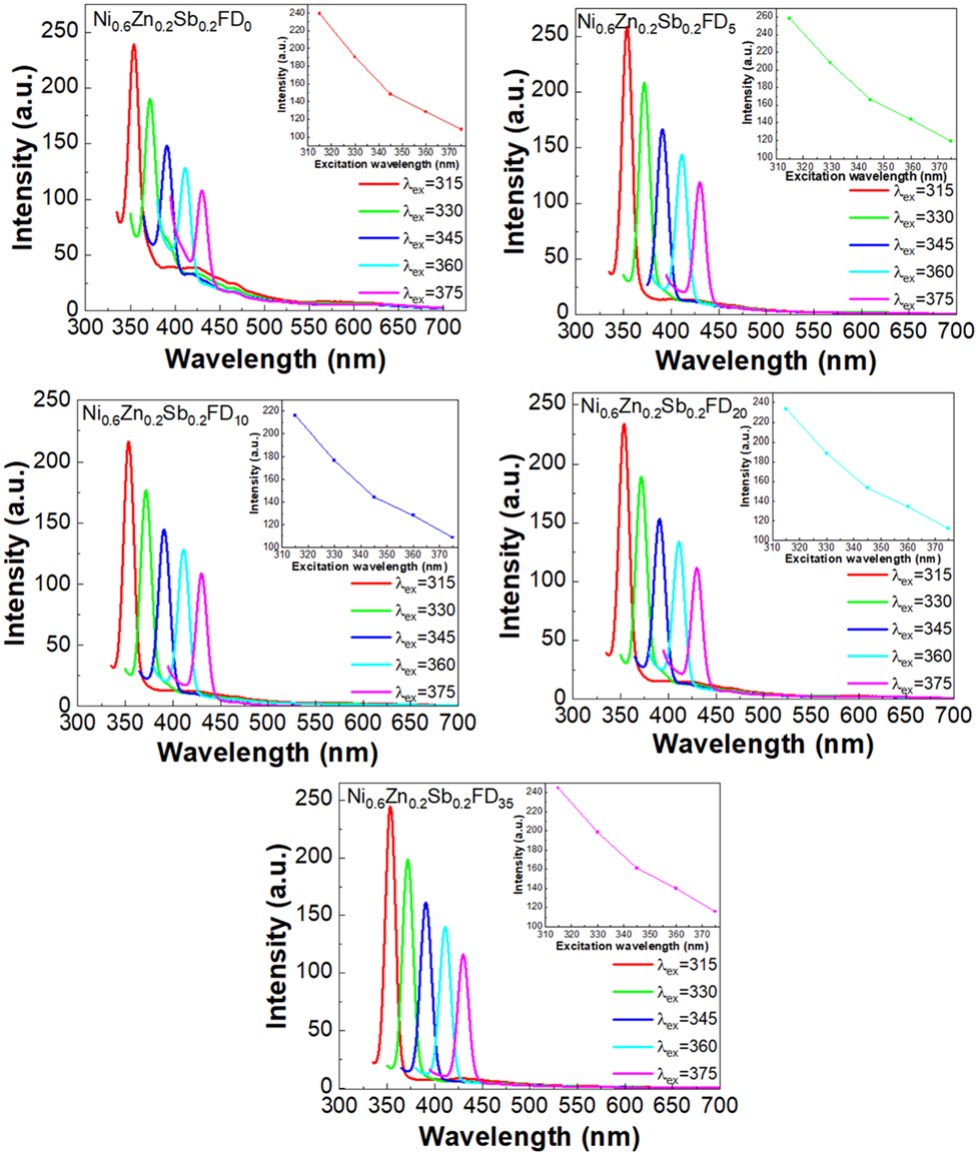
PL curves of the uncapped and capped Ni_0.6_Zn_0.2_Sb_0.2_FD at different excitation wavelengths.

Photoluminescence spectroscopy is a technique used to study the optical properties of nanoparticles and investigate the interstitial defects and the recombination process of electrons.^36^ PL studies on the synthesized nanoferrites were done at room temperature using 5 different excitation wavelengths (315, 330, 345, 360, and 370 nm) and are displayed in Fig. 9. As shown in Fig. 9, the spectra are divided into two regions: UV emission and visible emission. The intensity of the UV emission is higher compared to that of the visible emissions. This proves the excellent crystal quality of the synthesized nanoferrites and proposes their use in nano-optoelectronic devices.^37^ The peaks appearing in the UV region at all excitation wavelengths correspond to the near band edge emission, corresponding to the direct recombination of electrons trapped in oxygen vacancies with photogenerated holes.^38^ As shown in the inset of Fig. 9, the intensity of the peaks decreases with the increase in the excitation wavelength. This behavior is due to the enhancement of photogenerated electron–hole pairs at shorter wavelengths.^37^ Therefore, the synthesized samples can be utilized in light-emitting devices.^37^ Also, there is a red shift in the position of the peaks as a result of the selective particle size distribution in the synthesized nanoferrites due to polydispersion.^39^

**Fig. 10:**
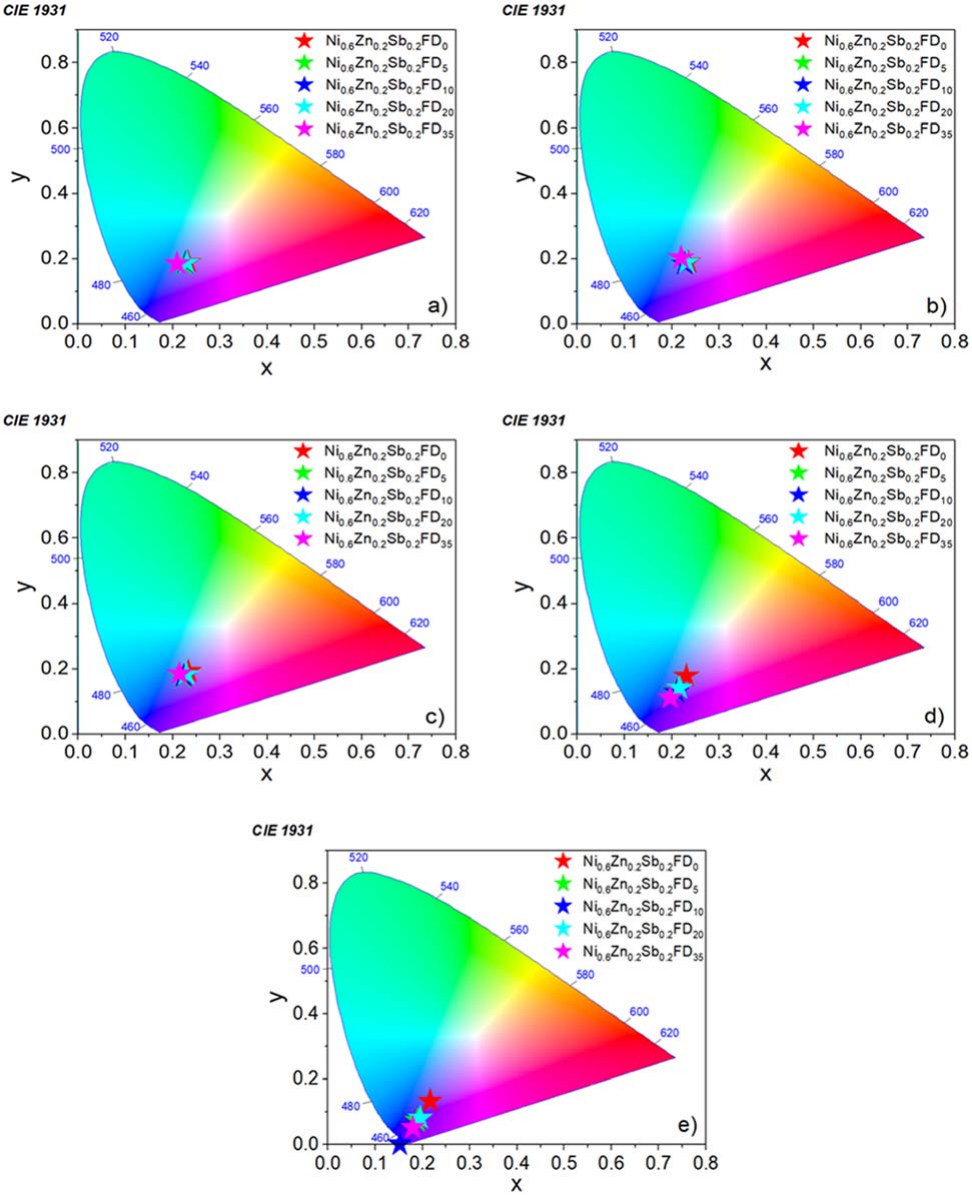
The CIE 1931 chromaticity diagrams of the uncapped and capped Ni_0.6_Zn_0.2_Sb_0.2_FD at different excitation wavelengths: (a) 315, (b) 330, (c) 345, (d) 360, and (e) 375 nm.

The CIE 1931 chromaticity diagrams of the uncapped and capped Ni_0.6_Zn_0.2_Sb_0.2_FD at different excitation wavelengths are depicted in Fig. 10. These diagrams are used to determine the color tenability and the luminescent behavior of the synthesized nanoferrites.^37^ This is done by calculating the color coordinates (*x*, *y*) of the emission spectra of the prepared samples. As displayed in the CIE diagrams, the coordinates of all samples are found in the blue region, signifying that the prepared nanoferrites can be utilized as a source of blue-chip light and near-UV white light-emitting diode devices.

### 3.5. Ni_0.6_Zn_0.2_Sb_0.2_FD_10_ adsorbent performance

#### 3.5.1 Optimization of adsorbent components

**Fig. 11:**
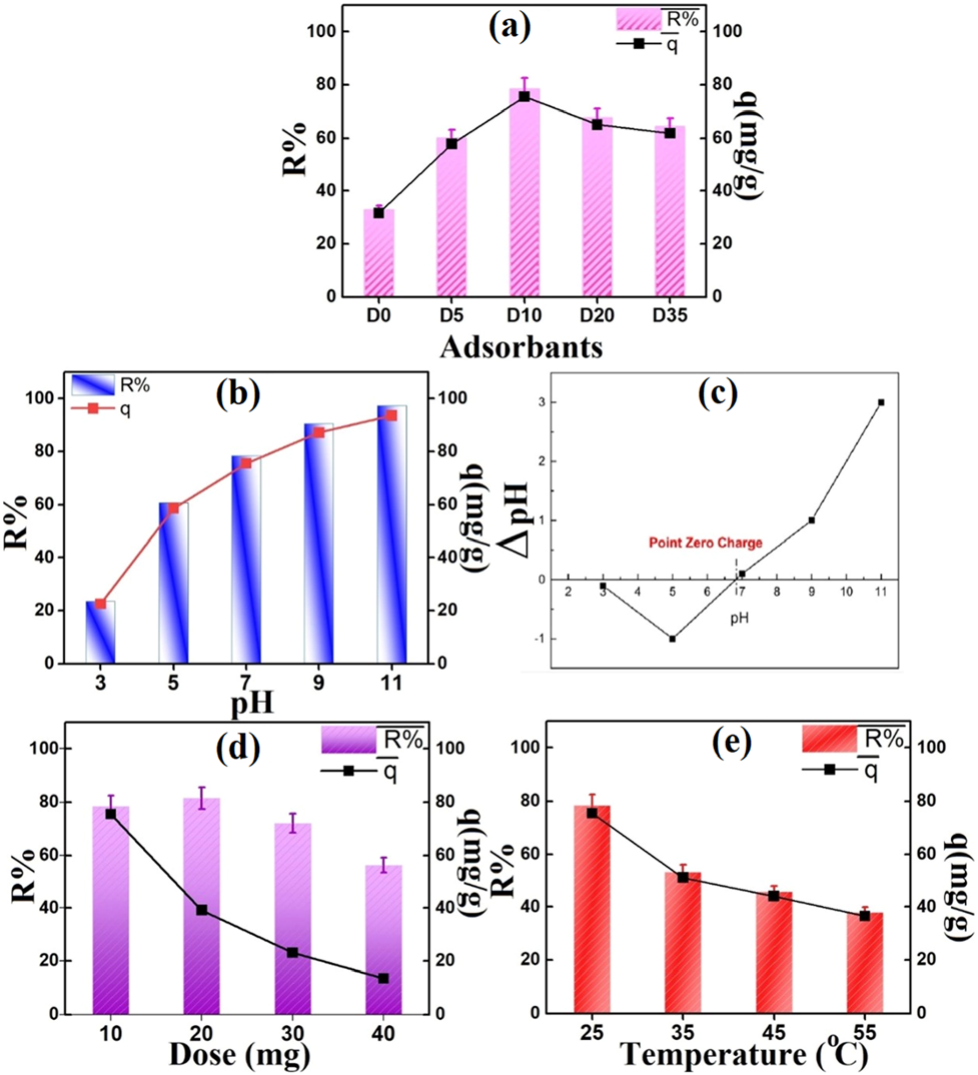
(a) Comparative study between the adsorption performance of the uncapped and capped Ni_0.6_Zn_0.2_Sb_0.2_FD, (b) impact of pH, (c) the zero-point charge diagram, (d) impact of Ni_0.6_Zn_0.2_Sb_0.2_FD_10_ dosage, and (e) impact of temperature on MV removal (Exp. conditions; MV = 10 mg L^−1^, adsorbent dose = 0.01 g, pH = 7, and an equilibrium time = 60 min).

To assess the adsorptive capability of dextrose monohydrate (*D*) in Ni_0.6_Zn_0.2_Sb_0.2_FD, a comparative test was conducted between the removal efficacy of MV by different incorporated concentrations of *D* at 0, 0.05, 0.1, 0.2, and 0.35 M, corresponding to *D*_0_, *D*_5_, *D*_10_, *D*_20_, and *D*_35_, as depicted in Fig. 11(a). On increasing *D* molar concentration in the adsorbent from 0 to 0.1 M, the MV removal efficacy and adsorption capacity boosted after 60 min from 32.9% and 31.6 mg g^−1^ to 78.5% and 75.5 mg g^−1^, respectively. This was attributed to the enhancement of adsorptive active sites. Whereas, the removal percentage and adsorption capacity declined to 64.3% and 61.9 mg g^−1^ in 0.35 M *D* after 60 min due to the blockage occurring at active sites in the adsorbent. Hence, the primary concentration of *D* was 0.1 M and exhibited higher values of *q*_max_ capacity and %*R* compared to the other samples, which were used for all studies. This is because Ni_0.6_Zn_0.2_Sb_0.2_FD_10_ sample has the largest size compared to the other samples, an *E*_g_ value = 2.98 eV, and an Urbach energy = 1.57 eV. The band gap energy and Urbach energy values indicate effective defect passivation and structural ordering, thus having a better removal efficiency and higher adsorption capacity compared to other samples.^27^ Moreover, a moderate dextrose monohydrate concentration decreases agglomeration and ensures an ordered surface structure having hydroxyl groups that form electrostatic interactions with the cationic MV dye.^40^

#### 3.5.2 The impact of different experimental factors on MV removal

To determine the best conditions for the removal of MV dye, several experimental factors were tested. These factors include the effect of pH, the effect of Ni_0.6_Zn_0.2_Sb_0.2_FD_10_ dosage, the effect of temperature, the effect of time, and the effect of MV initial concentration. Moreover, the recyclability test was carried out to study the ability to reuse and regenerate the adsorbent.

#### 3.5.3 Assessing the influence of the pH factor

The pH of the medium plays a crucial role in controlling the MV removal. It significantly affects the protonation or deprotonation of functional groups on adsorbent and adsorbate surfaces in solution. The absorbability of Ni_0.6_Zn_0.2_Sb_0.2_FD_10_ sample to MV was studied at pH ranges 3–11, as shown in Fig. 11(b). The MV removal percentage and adsorption capacity elevated from 23.4% and 22.5 mg g^−1^ to 98.7% and 95.9 mg g^−1^, corresponding to pH 3 to 11, respectively. This was confirmed by studying Ni_0.6_Zn_0.2_Sb_0.2_FD_10_ adsorbent surface charge as shown in Fig. 11(c). The decreased removal percent of MV cationic dye at low pHs may be due to electrostatic repulsion between the positively charged cationic MV dye and the positively charged Ni_0.6_Zn_0.2_Sb_0.2_FD_10_ surface (since at pH < 6.8, the surface is protonated).^41^ Besides, the high concentration of H^+^ ions in the medium competes with MV molecules for the occupation of Ni_0.6_Zn_0.2_Sb_0.2_FD_10_ active sites. At neutral pH (pH = pH_pzc_ = 6.8), the reduction in H^+^ ions in the medium diminishes the electrostatic repulsion and competition between protons and MV molecules on Ni_0.6_Zn_0.2_Sb_0.2_FD_10_ adsorbent, leading to an enhanced MV removal value at pH = 7.^42^ Whereas, at high pHs (pH > pH_pzc_), the significant shift in MV removal%, reaching 98.7% at pH 11.0, was attributed to the deprotonation that occurred to Ni_0.6_Zn_0.2_Sb_0.2_FD_10_, leaving a negative charge on Ni_0.6_Zn_0.2_Sb_0.2_FD_10_ surface that enhances the electrostatic attraction between the adsorbent and the cationic MV molecules. Moreover, Duynstee and Grunwald^43^ found that MV dye and OH^−^ ions interacted in the pH range of 9.0 to 13.0. Where these hydroxyl ions strike the MV molecule's core carbon atom, resulting in a base carbinol with a unique chemical structure that enhances the removal. Thus, we chose the optimal pH at 7 in our conducted experiments to ensure the role of Ni_0.6_Zn_0.2_Sb_0.2_FD_10_ surface on adsorption rather than OH^−^ ions.

#### 3.5.4 Assessing the influence of Ni_0.6_Zn_0.2_Sb_0.2_FD_10_ dose

The impact of varying the Ni_0.6_Zn_0.2_Sb_0.2_FD_10_ dosage on the MV adsorption was investigated. Fig. 11(d) shows the MV removal% at the dosage ranging from 10 to 40 mg Ni_0.6_Zn_0.2_Sb_0.2_FD_10_. The removal efficiency rose from 78.5% to 81.5% as the adsorbent dose increased from 10 to 20 mg. This rise can be explained by enhancing the Ni_0.6_Zn_0.2_Sb_0.2_FD_10_ surface area and the availability of adsorption sites. On the contrary, the decline in adsorption capacity from 72.1% to 56.3%, which corresponds to a 30 to 40 mg Ni_0.6_Zn_0.2_Sb_0.2_FD_10_ dose, might result from minimizing the surface area and hence the active adsorption sites due to particle aggregation.^44^ It was also shown that increasing Ni_0.6_Zn_0.2_Sb_0.2_FD_10_ dose reduced the quantity of adsorbed MV per unit mass of adsorbent from 75.5 to 13.5 mg g^−1^. With increasing adsorbent mass, *q*_e_ (mg g^−1^) falls due to a split in the concentration gradient between MV concentration in the solution and on the Ni_0.6_Zn_0.2_Sb_0.2_FD_10_ surface.^5^

#### 3.5.5 Assessing the influence of temperature

To investigate how temperature affects MV adsorption efficiency, tests were conducted at optimal circumstances and temperatures ranging from 25–55 °C, Fig. 11(e). The MV adsorption % reduced from 78.5% to 38.0% with rising temperatures of 25–55 °C. The adsorption efficiency reduced with higher temperatures due to various factors, including the MV molecules detaching from the Ni_0.6_Zn_0.2_Sb_0.2_FD_10_ surface and releasing into the solution. The changes and suppression in active sites, as well as the greater movement of MV molecules, occurred. These results and their interpretations are in agreement with findings reported in previous studies by Ahmadi A. *et al.*,^45^ Changzhen Li *et al.*,^46^ and Foroutan R. *et al.*^42^ Thus, the optimal temperature for MV elimination is 25 °C, which also yields the highest efficiency at 78.5%.

#### 3.5.6 Influence of contact time

**Fig. 12:**
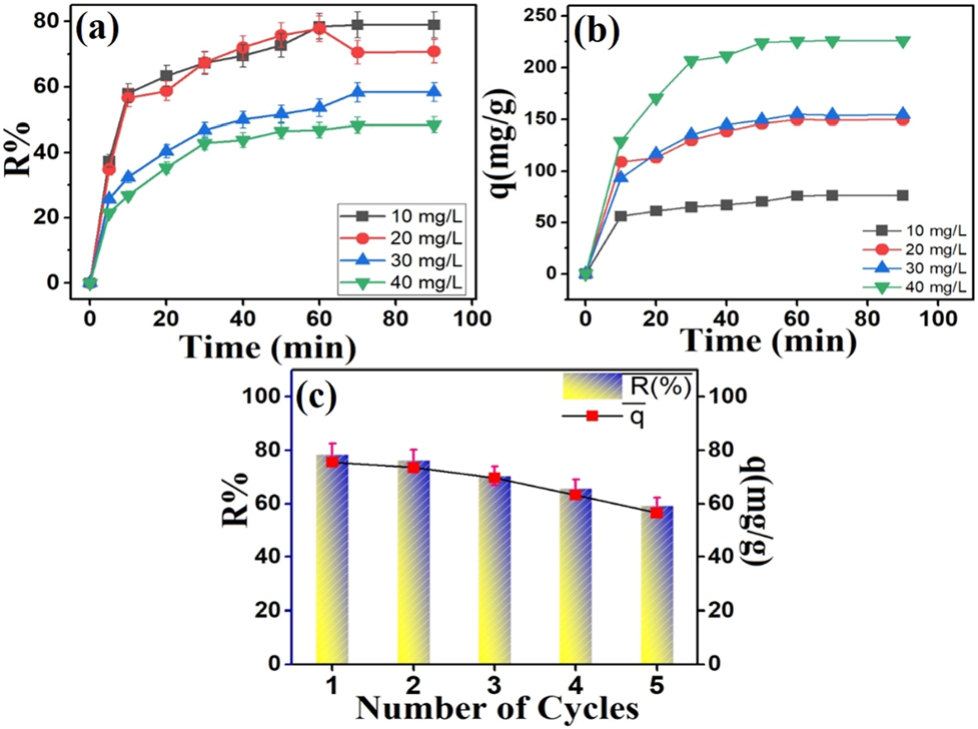
(a) Effect of contact time, (b) the effect of the initial concentration of MV, and (c) the recyclability test of Ni_0.6_Zn_0.2_Sb_0.2_FD_10_.

Fig. 12(a) illustrates the impact of contact time on the amount of adsorbed MV on the Ni_0.6_Zn_0.2_Sb_0.2_FD_10_ surface. It was observed that the initial stages of the adsorption process resulted in a swift rise in elimination, reaching 55.8 mg g^−1^, corresponding to a maximum removal% at 58.2% in 10 min. Then, after the adsorption of MV on Ni_0.6_Zn_0.2_Sb_0.2_FD_10_ was slowly increased until it attained the equilibrium time of 60 min, reaching a removal% and adsorption capacity of 78.5% and 75.5 mg g^−1^, respectively. The initial high MV removal% may be due to the abundance of unoccupied sites on the Ni_0.6_Zn_0.2_Sb_0.2_FD_10_ adsorbent. However, beyond the equilibrium time of 60 min, there is no noticeable removal effect since the number of available active sites was reduced due to saturation with MV dye molecules, as well as the electrostatic repulsive force between MV molecules was attained.^47^

#### 3.5.7 Influence of MV initial concentration

Fig. 12(b) portrays the MV dose-altering effect on the adsorption capability of Ni_0.6_Zn_0.2_Sb_0.2_FD_10_. After 60 min, MV removal efficiency declined from 78.5% with MV concentration of 10 mg L^−1^ to 46.8% with increasing MV concentration to 40 mg L^−1^. This was attributed to the higher MV concentrations causing more adsorption and adhesion of MV molecules on Ni_0.6_Zn_0.2_Sb_0.2_FD_10_ surface, leading to its active sites' obstruction.^48^ Besides, the experimental Ni_0.6_Zn_0.2_Sb_0.2_FD_10_ dosage condition was the same, even in higher MV concentrations.^44^ There was a reverse trend regarding the relationship between dye concentration and the adsorption capacity. Hence, the rise in dye concentration enhances the adsorption capacity as it increases the interaction between the adsorbate and the adsorbent. It was noticed in Fig. 12(b) that the spike in adsorption capacity from 75.5 to 225.3 mg g^−1^ when the MV concentration rose from 10 to 40 mg L^−1^ after 60 min. Increasing MV concentration can amplify its driving power, making the mass transfer easier, and allowing MV molecules to migrate from the bulk to the Ni_0.6_Zn_0.2_Sb_0.2_FD_10_ surface.^5^

### 3.6. Recyclability test

The ability to reuse and regenerate the adsorbent is an essential factor since it allows the conservation and sustainability of materials, provokes more applications, protects the environment, and is cost-effective.^49,50^ So, it is important to assess the recyclability test for Ni_0.6_Zn_0.2_Sb_0.2_FD_10_ sample. Therefore, the recyclability of Ni_0.6_Zn_0.2_Sb_0.2_FD_10_ sample was tested over 5 cycles. The adsorption capacity was measured after each cycle to assess any decline in performance. The recyclability test, depicted in Fig. 12(c), highlighted a decrease in MV adsorption from 78.5% and 75.6 mg g^−1^ to 69.3% and 66.7 mg g^−1^, respectively, after the 5th cycle. This outcome signified the stability of Ni_0.6_Zn_0.2_Sb_0.2_FD_10_ and demonstrated strong regenerative properties. Moreover, the slight decrease in MV adsorption and removal percentage after repeated usage and regeneration was due to the partial blockage and some collapse of Ni_0.6_Zn_0.2_Sb_0.2_FD_10_ active sites by MV molecules.^51^

### 3.7. Kinetic study

To explore the controlling mechanism of MV adsorption onto Ni_0.6_Zn_0.2_Sb_0.2_FD_10_, the experimental data were analyzed using pseudo-first-order (PFO), pseudo-second-order (PSO), Elovich models, and the intra-particle diffusion (IPD) model represented by eqn (20)–(23):^52,53^

20 ln(*q*_e_ − *q*_*t*_) = ln *q*_e_ −*k*_1_(*t*)(PFO),

21
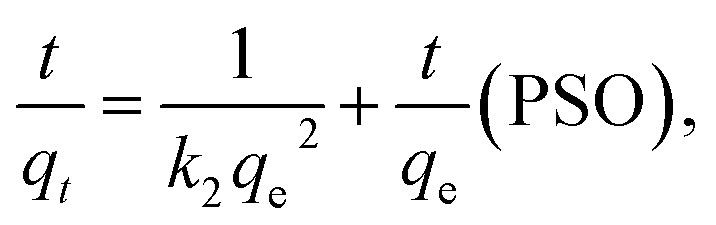
22
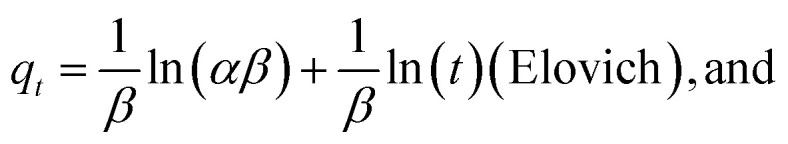

23 *q*_*t*_ = *k*_i_*t*^1/2^ + *C* (IPD).

where *q*_e_ represents the amount of MV that adsorbs onto Ni_0.6_Zn_0.2_Sb_0.2_FD_10_ at equilibrium, while *q*_*t*_ expresses the amount of MV adsorption at time *t*. *k*_1_ is the rate constant of the pseudo-first-order, and *k*_2_ is the rate constant pseudo-second-order. *α* and *β* are Elovich coefficients that represent the initial adsorption rate and the desorption coefficient, respectively, and also relate to the extent of surface coverage and activation energy for chemisorption. *K*_i_ is the IPD constant (mg g^−1^ min^0.5^), and *C* is the intercept (mg g^−1^).

**Fig. 13:**
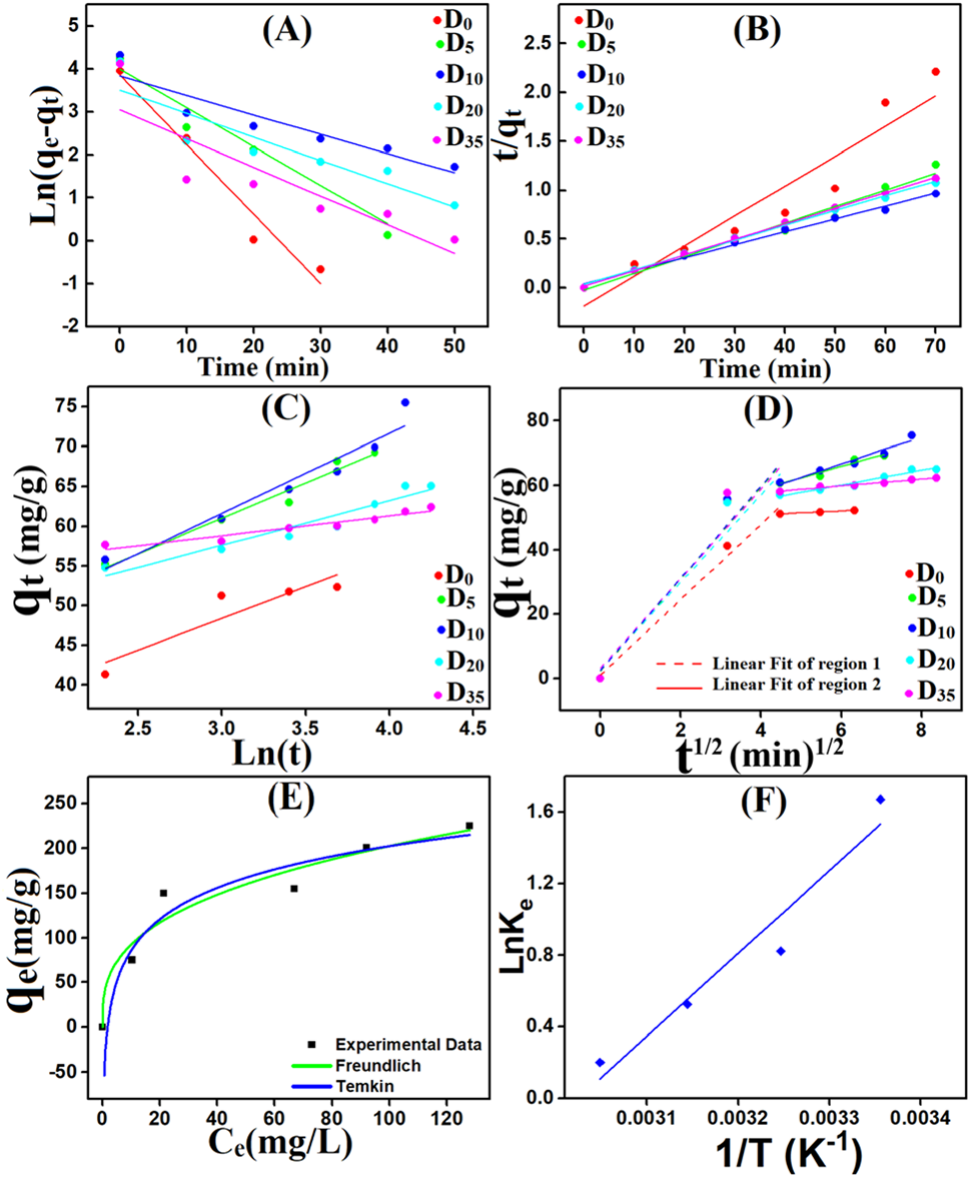
The linear relationships derived from: (a) pseudo-first-order kinetic plots, (b) pseudo-second-order kinetic plots, (c) the Elovich model, (d) the IPD model, *D*_0_, *D*_5_, *D*_10_, *D*_20_, *D*_35_ are dextrose concentration in Ni_0.6_Zn_0.2_Sb_0.2_FD. and (e) fitting representations of non-linear isotherm models for the adsorption of MV on Ni_0.6_Zn_0.2_Sb_0.2_FD_10_, and (f) van't Hoff's plot for the adsorption of MV onto Ni_0.6_Zn_0.2_Sb_0.2_FD_10_.

The linear relationships derived from the previously mentioned models are presented in Fig. 13(a–d) and Table 3. The removal of MV by uncapped Ni_0.6_Zn_0.2_Sb_0.2_FD_0_ is best fitted by PFO (*R*^2^ = 0.95). Whereas, the adsorption of MV onto capped Ni_0.6_Zn_0.2_Sb_0.2_FD by different dextrose concentrations (*D*_5_, *D*_10_, *D*_20_, *D*_35_) is well represented by the PSO model. This is revealed by the high *R*^2^ values (*R*^2^ = 0.99). Capping nano-ferrite with dextrose frequently changes the adsorption kinetics, from simple surface-limited physisorption to more sophisticated, chemically driven adsorption processes. The modified surface chemistry of capped Ni_0.6_Zn_0.2_Sb_0.2_FD adsorbs the MV molecules through strong interactions such as surface complexation, covalent bond formation, and electron sharing with MV molecules.^54^ Furthermore, a close similarity was observed between *q*_exp_ and *q*_cal_. Additionally, according to the Elovich model, the rate of adsorption *α* for MV was significantly higher than the rate of desorption *β*, supporting the reliability of the pseudo-second-order kinetic model.^55^ As shown in Fig. 13(d), two linear regions exist in all samples, indicating the presence of multiple stages in the adsorption process. Table 3 shows that the *k*_i_ values of region one are greater than those of region two. The first region indicates the rapid diffusion stage with external surface adsorption of MV dye, while the second region reveals a slow adsorption stage associated with the intraparticle diffusion of MV within the pores of the adsorbents. Moreover, the non-zero values of *C* indicate that the intra-particle diffusion is not the only rate-determining step.

**Table 3:** The derived kinetic parameters of the methyl violet adsorption onto Ni_0.6_Zn_0.2_Sb_0.2_FD

Kinetic models and parameters		*D*_0_	*D*_5_	*D*_10_	*D*_20_	*D*_35_
Experimental, *q*_exp_ (mg g^−1^)		31.60	57.80	75.50	65.10	61.90

**Pseudo 1st order**						
*R* ^2^		0.95	0.90	0.84	0.79	0.70
Calculated, *q*_cal_ (mg g^−1^)		47.60	54.65	46.22	33.49	21.29
*k*_1_		0.162	0.090	0.045	0.055	0.067

**Pseudo 2nd orde**r						
*R* ^2^		0.90	0.97	0.99	0.99	0.99
Calculated, *q*_cal_ (mg g^−1^)		32.52	58.31	75.41	66.05	62.58
*k*_2_		−0.005	−0.011	0.004	0.007	0.017

**Elovich**						
*α* (mg g^−1^ min^−1^)		170.99	432.58	231.92	8480.82	2.4 × 10^9^
*β* (g mg^−1^)		0.13	0.11	0.10	0.18	0.40
*R* ^2^		0.76	0.96	0.92	0.91	0.90

**IPD**						
Region 1	*C* (mg g^−1^)	0.94	2.25	2.35	2.66	3.07
	*k*_i_ (mg g^−1^ min^0.5^)	11.76	14.33	14.38	13.62	13.97
	*R* ^2^	0.98	0.92	0.91	0.88	0.84
Region 2	*C* (mg g^−1^)	48.76	44.81	41.73	46.56	53.56
	*k*_i_ (mg g^−1^ min^0.5^)	0.56	3.52	4.16	2.27	1.06
	*R* ^2^	0.99	0.92	0.94	0.95	0.97

### 3.8. Adsorption isotherm

Adsorption isotherm models play a fundamental role in describing the interactive behavior between the adsorbate and adsorbent. They are crucial for investigating the mechanisms of adsorption, the properties of the surface, and the affinity of the adsorbent.^56^ The non-linear isotherm models, including the Freundlich and Temkin, were employed. Their detailed descriptions are provided in the supplementary data (Text S1). The non-linear fit of these models for the adsorption of MV on Ni_0.6_Zn_0.2_Sb_0.2_FD_10_ is presented in Fig. 13(e). Also, the parameters obtained from the non-linear isotherm models are presented in Table 4. The adsorption behavior is best fit to the Temkin non-linear isotherm of correlation, *R*^2^ = 0.94. This fitting indicates that the adsorption process is dependent on the heat of adsorption (*b*_T_). When the *b*_T_ is a positive value, the adsorption process is exothermic. It is known from Temkin behavior that chemisorption often has typical adsorption energies, 
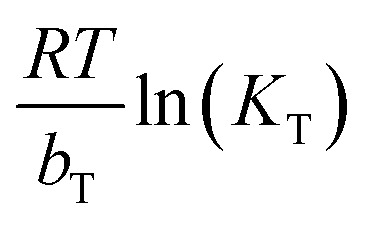
, ranging from 8–16 kJ mol^−1^, while physisorption has typical energies less than −40 kJ mol^−1^.^57^ Herein, the typical adsorption energy of MV on Ni_0.6_Zn_0.2_Sb_0.2_FD_10_, 
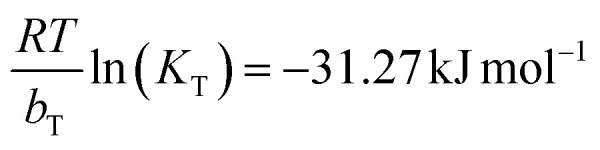
, besides, *b*_t_ value is less than 80 kJ mol^−1^ (ref. 57) which confirms the physisorption interaction. Moreover, the Freundlich model provides a good explanation for adsorption behavior, owing to adjusted *R*^2^ = 0.937. Since *n* > 1, the adsorption is favorable and occurs in terms of physical behavior. The heterogeneity factor 
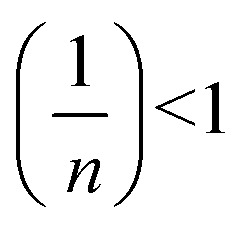
, which provides high surface heterogeneity. The higher value of *K*_f_ reflects the greater adsorption potential.

**Table 4:** The parameters derived from the non-linear isotherm models for methyl violet adsorption onto Ni_0.6_Zn_0.2_Sb_0.2_FD_10_

Isotherm models and parameters	Value
**Freundlich**	
*K*_F_	42.192
*n*	2.936
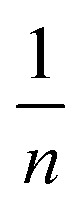	0.341
*R* ^2^	0.937

**Temkin**	
*b*_T_ (J mol^−1^)	48.827
*K*_T_ (L mg^−1^)	0.540
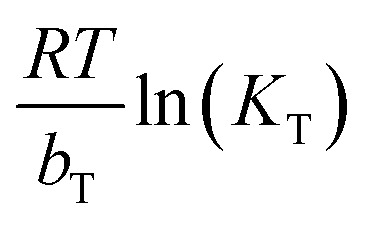	−31.27
*R* ^2^	0.940

### 3.9. Thermodynamic study

Thermodynamic parameters were analyzed to understand the mechanisms, spontaneity, energetics, and feasibility of MV adsorption processes during the temperature range 25–55 °C. The van't Hoff formula was applied to calculate Gibbs' free energy change (Δ*G*), enthalpy change (Δ*H*), and entropy change (Δ*S*), eqn (24)–(26).^58–61^24
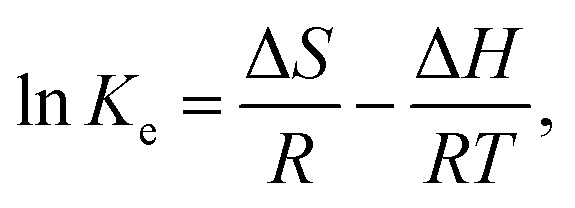

25 Δ*G* = Δ*H* − *T*Δ*S*,

26
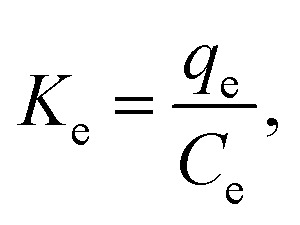
where *K*_e_ is the equilibrium constant, *q*_e_ is the amount of adsorbed MV per gram of the adsorbent at equilibrium (mg g^−1^), *C*_e_ is the concentration of MV at equilibrium, *R* is the ideal gas constant, and *T* is the temperature, the van't Hoff plot was depicted in Fig. 13(f).

The Δ*G* values for the adsorption process of MV onto Ni_0.6_Zn_0.2_Sb_0.2_FD_10_ at different temperatures were negative, −3.79, −2.63, −1.46, and −0.30 kJ mol^−1^ for 25, 35, 45, and 55 °C, suggesting that adsorption occurred feasibly and spontaneously. Δ*H* value (−38.5 kJ. mol^−1^) suggests that the adsorption process of MV onto Ni_0.6_Zn_0.2_Sb_0.2_FD_10_ is exothermic. It is known that the Δ*H* parameter may be used to categorize adsorption as physical when Δ*H* < 84 kJ mol^−1^, or chemical when 184 < Δ*H* < 420 kJ mol^−1^.^41^ In our study, the Δ*H* value indicates that MV adsorption is a physical process, consistent with the Δ*G* parameter values, Fig. 13(f). Additionally, the negative entropy change Δ*S*° (−116.47 J mol^−1^ K^−1^) is evidence of a reduction in collision randomness between MV molecules and Ni_0.6_Zn_0.2_Sb_0.2_FD_10_ interface.^42^

### 3.10. The plausible MV elimination mechanism

**Fig. 14:**
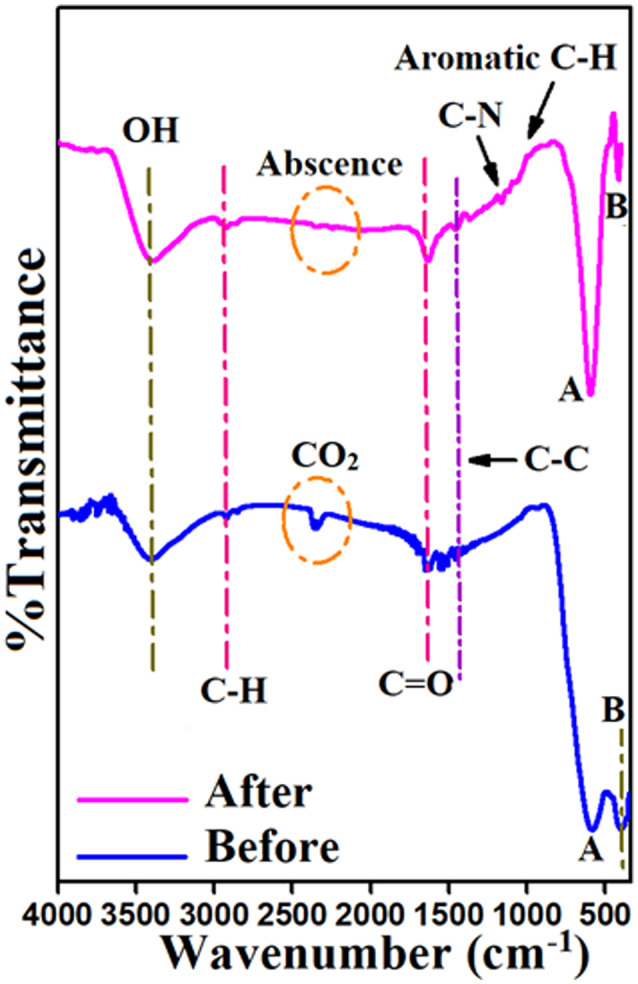
FTIR before and after MV adsorption on Ni_0.6_Zn_0.2_Sb_0.2_FD_10_ surface.

To clarify the mechanism of MV adsorption on Ni_0.6_Zn_0.2_Sb_0.2_FD_10_, FTIR was analyzed before and after MV adsorption. According to Fig. 14, the metal–oxygen (M–O) stretching vibrations of tetrahedral (A) sites were shown at the band with strong absorption and a greater wavenumber at 587 cm^−1^, while the M–O octahedral (B) site vibrations in Ni_0.6_Zn_0.2_Sb_0.2_FD_10_ were depicted at the reduced intensity absorbance band with a lower wavenumber at 400 cm^−1^. As a result of these M–O spectra, the spinel structure has been confirmed.^20^ The signature bands of OH, aliphatic asymmetric C–H, C=O, and C–C were recorded at 3400 cm^−1^, 2915 cm^−1^, 1655 cm^−1^, and 1473 cm^−1^ stretching frequencies. While the bending character vibration of C–H appeared at 1361 cm^−1^.^62^ The observed band at 2360 cm^−1^ is attributed to CO_2_ present in the atmosphere. Using the Ni_0.6_Zn_0.2_Sb_0.2_FD_10_ to adsorb cationic MV dye caused a dramatic shift in the position and intensity of the spectrum's peaks, Fig. 14. The bands of O–H, C=O, and C–C apparently shifted from 3400 cm^−1^, 1655 cm^−1^, and 1473 cm^−1^ to 3393 cm^−1^, 1635 cm^−1^, and 1458 cm^−1^ before and after loading MV, respectively. Besides, the stretching and bending vibrations of C–H migrated from 2915 cm^−1^ and 1361 cm^−1^ to 2926 cm^−1^ and 1368 cm^−1^, respectively. Furthermore, metal-oxo bond shifting from 587 cm^−1^ and 400 cm^−1^ to 594 cm^−1^ and 416 cm^−1^ occurred, respectively. The additional stretching vibration band appeared at 1164 cm^−1^, corresponding to C–N in MV. The absence of the 2360 cm^−1^ band indicated that CO_2_ was poorly adsorbed, and MV physically removed or inhibited it from adhering around. Multiple out-of-plane C–H bending vibration peaks were found between 600 and 1000 cm^−1^, indicating an aromatic structure.^47^ In conclusion, there is a hybrid adsorption mechanism that is defined by the concurrent presence of both chemisorption and physisorption processes. This is consistently supported by evidence from the spectroscopic (FT-IR) shifts, the fitted kinetic and isotherm models, and the estimated thermodynamic parameters. MV elimination on Ni_0.6_Zn_0.2_Sb_0.2_FD_10_ surfaces could be attributed to physical pathways, including coordination, hydrogen bonding, van der Waals forces, and hydrophobic electrostatic contact. This elimination route occurred through interaction between M–O, –OH, C=O, C–C, and C–O binding sites on the Ni_0.6_Zn_0.2_Sb_0.2_FD_10_ adsorbent with nitrogen, and aromatic rings on the MV molecule's surface. The FTIR analysis following MV adsorption supported this conclusion. Additionally, the isotherms of MV adsorption onto Ni_0.6_Zn_0.2_Sb_0.2_FD_10_ surfaces also demonstrate physical behavior of these adsorption processes. Temkin's isotherm model is the best for describing the physisorption mechanism as *b*_t_ < 80 kJ mol^−1^. It suggests that this sort of adsorption includes weak van der Waals forces, resulting in the creation of multilayers on the Ni_0.6_Zn_0.2_Sb_0.2_FD_10_ surface.^48^ This was aligned with the thermodynamic Δ*H* value (−38.5 kJ mol^−1^) that proved the physisorption.^41^ Whereas, the most suitable kinetic model for MV adsorption on capped Ni_0.6_Zn_0.2_Sb_0.2_FD_10_ was pseudo-second order. Since dextrose capping of Ni_0.6_Zn_0.2_Sb_0.2_F alters the surface chemistry of the nano-ferrite adsorbent. This change is responsible for the chemisorption MV removal on Ni_0.6_Zn_0.2_Sb_0.2_FD_10_ surface. This was confirmed by the Elovich model, which computed parameters *α* and *β* as the adsorption rate is higher than the desorption rate (*α* >*β*).^55^

### 3.11. Comparative study

Table 5 shows that Ni_0.6_Zn_0.2_Sb_0.2_FD_10_ has a strong affinity for MV molecules with excellent adsorption efficiency in a short equilibrium time in comparison with other adsorbents. Ni_0.6_Zn_0.2_Sb_0.2_FD_10_ attained high adsorption capability in neutral circumstances (pH = 7) and prevented solution neutralization after adsorption. The prior studies required a mildly acidic or weak and strong basic media to achieve a considerable removal efficiency.^42,47,63–67^ Furthermore, Ni_0.6_Zn_0.2_Sb_0.2_FD_10_ reached high removal percent and adsorption capacity by 78.5% and 75.5 mg g^−1^, respectively, for 10 ppm MV, neutral pH, 20 mg adsorbent dosage in equilibrium time = 60 min. This significant MV removal of Ni_0.6_Zn_0.2_Sb_0.2_FD_10_ is higher than previously fabricated adsorbents, as depicted in Table 5.

**Table 5:** The excellent adsorption efficiency of MV on Ni_0.6_Zn_0.2_Sb_0.2_FD_10_ with a fast equilibrium period in comparison with other capped and non-capped ferrites

Adsorbent	*q*_max_ (mg g^−1^)	Experimental conditions (adsorbent amount, pH, dye concentration, contact time)	References
MMT/GO/CoFe_2_O_4_	97.26	1 g L^−1^, 8, 10 mg L^−1^, 40 min	42
Nickel ferrite	380	25 mg, 11, 10 mg L^−1^	47
AC/CoFe_2_O_4_	83.90	3 g L^−1^, 8, 10 mg L^−1^, 40 min	63
NiFe_2_O_4_-SDS	5.195	0.1 g, 5.5, 15 mg L^−1^, 25 min	64
Ni_0.5_Zn_0.5_Fe_2_O_4_ nanocomposite	500	0.06 g, 7, 20 mg L^−1^, 120 min	65
NiCuZnFe_2_O_4_ ferrite-biochar composite	325.5	30 mg, 8, 250 mg L^−1^, 120 min	66
SDS-coated GS nanoparticles	174.2	10 mg, 3, 15 mg L^−1^, 5 min	67
Ni_0.6_Zn_0.2_Sb_0.2_FD_10_	75.5	20 mg, 7, 10 ppm, 60 min	This study

## 4. Conclusion

Different concentrations of dextrose monohydrate were utilized to cap Ni_0.6_Zn_0.2_Sb_0.2_Fe_2_O_4_. Several optical parameters were calculated, and the effect of capping agent concentration was studied. It was found that Ni_0.6_Zn_0.2_Sb_0.2_FD_10_ had the highest transmittance, making it suitable for optoelectronic applications. The band gap energy varied between 2.71 and 3.05 eV, where Ni_0.6_Zn_0.2_Sb_0.2_FD_5_ sample registered the lowest value, while Ni_0.6_Zn_0.2_Sb_0.2_FD_35_ attained the highest value. The refractive index value ranged between 2.21 and 2.39 for all samples. Moreover, the PL studies shed light on the potential use of the synthesized nanoferrites in blue-chip light-emitting diode, by studying their chromaticity diagram. Moreover, the adsorption activity of the Ni_0.6_Zn_0.2_Sb_0.2_FD_10_ against MV was examined. It showed the best performance with a removal efficiency of 78.5% at pH = 7, 60 min for 10 ppm MV by 10 mg Ni_0.6_Zn_0.2_Sb_0.2_FD_10_. The maximum adsorption capacity reached 75.5 mg g^−1^ at equilibrium time. Moreover, Ni_0.6_Zn_0.2_Sb_0.2_FD_10_ was stable and reusable for up to five cycles. MV adsorption efficiency is substantially improved by the synergistic combination of two removal pathways. The first one is a physical route including coordination, hydrogen bonding, van der Waals forces, hydrophobic electrostatic contact, and/or pore filling. While the second route is chemical, involving covalent and hydrogen bonding. The MV adsorption study on Ni_0.6_Zn_0.2_Sb_0.2_FD_10_ matches well with pseudo-second-order and follows the Temkin non-linear isotherm model. Furthermore, MV Adsorption occurred spontaneously and exothermically at temperatures ranging from 25 to 55 °C.

## Author contributions

All the authors contributed to this study. Nourhan Mohamed Gaber: adsorption studies performance, data collection, data curation, data analysis, formal analysis, and writing. Leen W. El Khatib: sample preparation, data collection, data curation, data analysis, formal analysis, and writing. Amani Aridi: review and editing. Alaa M. Abdallah: data curation, formal analysis, and review and editing. Gehan M. El-Subruiti: conceptualization, review and supervision. Mirna Omar: adsorption studies performance, data collection, data analysis, and writing. Sarah Omar: adsorption studies performance, data collection, data analysis, and writing. Ramadan Awad: conceptualization, review and supervision. All authors reviewed and approved the manuscript.

## Conflicts of interest

The authors declare that they have no competing interests relevant to this article's content.

## Supplementary Material

RA-016-D5RA08391E-s001

## Data Availability

The datasets supporting the findings of this study are available within the article and its supplementary information (SI) files. Supplementary information (SI) is available. See DOI: https://doi.org/10.1039/d5ra08391e.

## References

[cit1] Akbari A., Abbasi H., Shafiee M., Baniasadi H. (2024). Int. J. Biol. Macromol..

[cit2] Faizal A. N. M., Putra N. R., Zaini M. A. A. (2023). Part. Sci. Technol..

[cit3] Bonetto L. R., Ferrarini F., de Marco C., Crespo J. S., Guégan R., Giovanela M. (2015). J. Water Process Eng..

[cit4] Mohd Faizal A. N., Putra N. R., Abdul Aziz A. H., Agi A., Ahmad Zaini M. A. (2024). J. Clean. Prod..

[cit5] Sadiku M., Selimi T., Berisha A., Maloku A., Mehmeti V., Thaçi V., Hasani N. (2022). Toxics.

[cit6] Al-Degs Y. S., El-Barghouthi M. I., El-Sheikh A. H., Walker G. M. (2008). Dyes Pigments.

[cit7] Gong Z.-X., Steven M., Chen Y.-T., Huo L.-Z., Xu H., Guo C.-F., Yang X.-J., Wang Y.-X., Luo X.-P. (2024). RSC Adv..

[cit8] Gaber N. M., El-Subruiti G. M., Omer A. M., Eltaweil A. S. (2024). Surf. Interfaces.

[cit9] Soufi A., Hajjaoui H., Elmoubarki R., Abdennouri M., Qourzal S., Barka N. (2021). Appl. Surf. Sci. Adv..

[cit10] Javed R., Sajjad A., Naz S., Sajjad H., Ao Q. (2022). Int. J. Mol. Sci..

[cit11] Sidhu A. K., Verma N., Kaushal P. (2022). Front. Nanotechnol..

[cit12] Khatib L. W. E., Abdallah A. M., Noun M., Ghouch N. E., Younes G. O., Awad R. (2025). J. Mater. Sci. Mater. Electron..

[cit13] Javed R., Zia M., Naz S., Aisida S. O., ul Ain N., Ao Q. (2020). J. Nanobiotechnology.

[cit14] Prabhakaran T., Mangalaraja R. V., Denardin J. C., Varaprasad K. (2018). J. Mater. Sci. Mater. Electron..

[cit15] Javed R., Usman M., Tabassum S., Zia M. (2016). Appl. Surf. Sci..

[cit16] Kamari H. M., Naseri M. G., Saion E. B. (2014). Metals.

[cit17] Goodarz Naseri M., Saion E., Khalil Zadeh N. (2013). Int. Nano Lett..

[cit18] Goldstein J. I. , Newbury D. E., Michael J. R., Ritchie N. W. M., Scott J. H. J. and Joy D. C., Scanning Electron Microscopy and X-Ray Microanalysis, Springer, 2017

[cit19] Watts J. F. and Wolstenholme J., An Introduction to Surface Analysis by XPS and AES, John Wiley & Sons, 2019

[cit20] Yassine R., Abdallah A. M., Hassan R. S., Yaacoub N., Awad R., Bitar Z. (2023). J. Nanoparticle Res..

[cit21] Abdallah A. M., Awad R. (2022). Phys. B Condens. Matter.

[cit22] Kumari C., Dubey H. K., Naaz F., Lahiri P. (2020). Phase Transit..

[cit23] Talebi R., Nasiri M., Rahnamaeiyan S. (2016). J. Mater. Sci. Mater. Electron..

[cit24] Chehade W., Basma H., Abdallah A. M., Sayed Hassan R., Awad R. (2022). Ceram. Int..

[cit25] Al-Hammadi A., Khoreem S. (2023). Biointerface Res. Appl. Chem..

[cit26] Sagar A., Bhardwaj S., Gupta A., Tripathi H., Shukla R. (2024). Interactions.

[cit27] Salih S. J., Mahmood W. M. (2023). Heliyon.

[cit28] Noreen S., Hussain A. (2023). Opt. Mater..

[cit29] Chebbi M., Mansouri S., Hcini S., Ghiloufi I., Mimouni A., Mir L. E. (2024). J. Mol. Struct..

[cit30] Hashim A. (2021). J. Inorg. Organomet. Polym. Mater..

[cit31] Soliman T. S., Vshivkov S. A. (2019). J. Non-Cryst. Solids.

[cit32] Pawar C. S., Gujar M. P., Mathe V. L. (2017). J. Supercond. Nov. Magn..

[cit33] Lemziouka H., Boutahar A., Moubah R., Omari L. H., Bahhar S., Abid M., Lassri H. (2020). Vacuum.

[cit34] Savithri Vatsalya V. L., Sundari G. S., Sridhar Ch. S. L. N., Prasanna I. L., Lakshmi Ch. S. (2022). J. Lumin..

[cit35] Assim E. M. (2008). J. Alloys Compd..

[cit36] Sharma R., Thakur P., Kumar M., Barman P. B., Sharma P., Sharma V. (2017). Ceram. Int..

[cit37] Abdallah A. M., Noun M., Awad R. (2021). Appl. Phys. A.

[cit38] Debnath S., Das R. (2020). J. Mol. Struct..

[cit39] Osman M. A., Abd-Elrahim A. G. (2018). Opt. Mater..

[cit40] Ivanets A., Prozorovich V., Roshchina M., Kouznetsova T., Budeiko N., Kulbitskaya L., Hosseini-Bandegharaei A., Masindi V., Pankov V. (2021). Chem.–Eng. J..

[cit41] Foroutan R., Mohammadi R., Ahmadi A., Bikhabar G., Babaei F., Ramavandi B. (2022). Chemosphere.

[cit42] Foroutan R., Mohammadi R., MousaKhanloo F., Sahebi S., Ramavandi B., Kumar P. S., Vardhan K. H. (2020). Adv. Powder Technol..

[cit43] Duynstee E. F. J., Grunwald E. (1959). J. Am. Chem. Soc..

[cit44] Eltaweil A. S., Mohamed Gaber N., El-Subruiti G. M., Omer A. M. (2024). J. Mol. Liq..

[cit45] Ahmadi A., Foroutan R., Esmaeili H., Peighambardoust S. J., Hemmati S., Ramavandi B. (2022). Mater. Chem. Phys..

[cit46] Li C., Dong Y., Yang J., Li Y., Huang C. (2014). J. Mol. Liq..

[cit47] Debnath S., Das R. (2023). Ceram. Int..

[cit48] Gupta S. V., Kulkarni V. V., Ahmaruzzaman Md. (2024). Colloids Surf. Physicochem. Eng. Asp..

[cit49] Hassan N., Shahat A., El-Didamony A., El-Desouky M. G., El-Bindary A. A. (2020). J. Mol. Struct..

[cit50] Almahri A., Abou-Melha K. S., Katouah H. A., Al-bonayan A. M., Saad F. A., El-Desouky M. G., El-Bindary A. A. (2023). J. Mol. Struct..

[cit51] Adel M., Ahmed M. A., Mohamed A. A. (2021). Environ. Nanotechnol. Monit. Manag..

[cit52] Abd El-Monaem E. M., Hosny M., Eltaweil A. S. (2024). Chem. Eng. Sci..

[cit53] Omer A. M., El-Sayed M., Abd El-Monaem E. M., El-Subruiti G. M., Eltaweil A. S. (2023). Int. J. Biol. Macromol..

[cit54] Aridi A., Rmeid S., El Sayed M. Y., Habanjar K., El-Subruiti G. M., Abdel Rahman E. M., Khalil W. F., Awad R., Gaber N. M. (2026). Mater. Sci. Eng. B.

[cit55] Eltaweil A. S., Awad A. E., Abd El-Monaem E. M., Shaker A. M., El-Subruiti G. M. (2025). J. Mol. Struct..

[cit56] Açıkyıldız M., Gürses A., Güneş K., Yalvaç D. (2015). Appl. Surf. Sci..

[cit57] Rahangdale D., Kumar A. (2018). Carbohydr. Polym..

[cit58] Zghal S., Jedidi I., Cretin M., Cerneaux S., Abdelmouleh M. (2023). Materials.

[cit59] Ali N. S., Jabbar N. M., Alardhi S. M., Majdi H. S., Albayati T. M. (2022). Heliyon.

[cit60] Kul A. R., Aldemir A., Alkan S., Elik H., Çalışkan M. (2019). Environ. Res. Technol..

[cit61] Alharby N. F., Almutairi R. S., Mohamed N. A. (2021). Polymers.

[cit62] Padwal Y., Chauhan R., Jeet Chaudhary I., Late D. J., Ashokkumar M., Gosavi S. (2025). Energy Adv..

[cit63] Foroutan R., Mohammadi R., Ramavandi B. (2019). Environ. Sci. Pollut. Res..

[cit64] Alizadeh N., Mahjoub M. (2017). J. Nanoanalysis.

[cit65] Afkhami A., Sayari S., Moosavi R., Madrakian T. (2015). J. Ind. Eng. Chem..

[cit66] Mehta D., Dave P. N., Vijay Kumar V. (2025). RSC Adv..

[cit67] Tiwari A. N., Tapadia K., Thakur C. (2022). Water Sci. Technol..

