# Supplementary material for: Influence of dextrose monohydrate on the optical properties and adsorption activity of Ni0.6Zn0.2Sb0.2Fe2O4 nanoferrites: towards multifunctional applications
Source: RSC Adv. 2026 Jan 21;16(5):4575–89. doi: 10.1039/d5ra08391e (PMC12821126; doi:10.1039/d5ra08391e)
Supplement: RA-016-D5RA08391E-s001 [file RA-016-D5RA08391E-s001.pdf]

Supplementary Information (SI) for RSC Advances.
This journal is © The Royal Society of Chemistry 2026

Influence of Dextrose Monohydrate on the Optical Properties and Adsorption Activity of $Ni_{0.6}Zn_{0.2}Sb_{0.2}Fe_2O_4$ Nanoferrites: Towards Multifunctional Applications

Nourhan Mohamed Gaber [1,*], Leen W. El Khatib[2], Amani Aridi[3], Alaa M. Abdallah[4], Gehan M. El-Subruiti[5], Mirna Omar[5,6], Sarah Omar[5,6], Ramadan Awad[7,8]

[1] Department of Medical Laboratories, Faculty of Applied Health Science Technology, Pharos University in Alexandria; Canal El Mahmoudia Street, Beside Green Plaza Complex 21648, Alexandria, Egypt

[2] Department of Chemistry, Faculty of Science, Beirut Arab University, Beirut, Lebanon

[3] Public health department, Faculty of Health Sciences, Modern University for Business and Science, Beirut, Lebanon

[4] Physics Department, Faculty of Science, Beirut Arab University, Beirut, Lebanon

[5] Chemistry Department, Faculty of Science, Alexandria University, Alexandria, Egypt

[6] Department of Mechanical Engineering, Universiti Teknologi PETRONAS (UTP), 32610 Bandar Seri Iskandar, Perak Darul Ridzuan, Malaysia

[7] Physics Department, Faculty of Science, Alexandria University, Alexandria, Egypt

[8] Department of Basic Sciences, Faculty of Computer Science and Artificial Intelligence, Pharos University in Alexandria, Alexandria, Egypt

*nourhan.gaber@pua.edu.eg

Text S1

3.8. Adsorption isotherm

The Freundlich isotherm is a complex model. This model considers multilayer adsorption on heterogeneous surfaces. The adsorption process is non-ideal and reversible with the presence of interaction between the adsorbed molecules. The non-linear equation of this model is described by Eq. (s1).[1]

$$q_e = K_F C_e^{1/n} \text{ (Freundlich)}, \tag{s1}$$

where K_F is the Freundlich constant and n is the adsorption intensity. The value of n gives an insight into the degree of non-linearity between the solution concentration and adsorption. If $n = 1$, then the adsorption is linear. If n < 1 indicates that the adsorption process occurs via chemical interactions; however, if n >1, then the adsorption takes place through a physical process.[2] Also, the Temkin isotherm model accounts for the adsorbate-adsorbent molecules. As an outcome of this interaction, the heat of adsorption of all molecules in the layer decreases linearly with coverage. Moreover, a uniform distribution of binding energy characterizes the adsorption.[3] The non-linear Temkin isotherm model is expressed in Eq. (s2).[4]

$$q_e = \frac{RT}{b_T} \ln{(K_T C_e)} \text{ (Temkin)}, \tag{s2}$$

where b_T is the coefficient related to the sorption heat (J.mol^{-1}), and K_T is the equilibrium binding constant (L.mg^{-1}). Typical adsorption energy, $\frac{RT}{b_T} \ln{(K_T)}$. T is the absolute temperature in K and R is the gas constant (8.314 J/mol. K).

References

1 A. S. Eltaweil, A. E. Awad, E. M. Abd El-Monaem, A. M. Shaker and G. M. El-Subruiti, *J. Mol. Struct.*, 2025, **1337**, 142143.
2 L. Yan, L. Qin, H. Yu, S. Li, R. Shan and B. Du, *J. Mol. Liq.*, 2015, **211**, 1074–1081.
3 P. Senthil Kumar, S. Ramalingam, C. Senthamarai, M. Niranjanaa, P. Vijayalakshmi and S. Sivanesan, *Desalination*, 2010, **261**, 52–60.
4 S. Liu, H. Ouyang and Z. Wang, *Mater. Res. Express*, 2022, **9**, 075003.
